# Beyond the more-is-better assumption: latent profiles of visual arts engagement, emotion regulation, and mental health among Chinese non-art-major university students

**DOI:** 10.3389/fpsyg.2026.1934081

**Published:** 2026-09-09

**Authors:** Qiong Ye, Kaiwen Ren, Da Wang, Xianwen Li, Mengting Qian

**Affiliations:** 1 School of Arts, Qinghai Nationalities University, Xining, China; 2 School of Smart Education, Jiangsu Normal University, Xuzhou, China; 3 Graduate School of Fine Arts, Hongik University, Seoul, Republic of Korea; 4 School of Fine Arts, Northwest Minzu University, Lanzhou, China; 5 Department of Education Management, Faculty of Education, East China Normal University, Shanghai, China

**Keywords:** emotion regulation, latent profile analysis, mental health, psychological distress, subjective well-being, visual arts engagement

## Abstract

Although visual arts engagement is widely regarded as beneficial for psychological health, more frequent participation may not always correspond to more favorable outcomes. This cross-sectional survey identified latent profiles of visual arts engagement among 586 Chinese non-art-major university students and examined their associations with subjective well-being, psychological distress, and emotion regulation. Latent profile analysis was based on four engagement dimensions: active creation, receptive appreciation, digital engagement, and social/learning involvement. Profile differences were tested using Welch’s ANOVA, Games–Howell *post hoc* comparisons, BCH sensitivity analyses, and parallel statistical indirect association models including cognitive reappraisal and expressive suppression. A four-profile solution was retained as a parsimonious person-centered representation of a graded engagement continuum, distinguishing very low, low, moderate, and high visual arts engagement. Students in the high-engagement profile reported the lowest subjective well-being and the highest depression, anxiety, stress, and overall psychological distress. Continuous-model sensitivity analyses indicated that profile membership did not provide significant explanatory value beyond continuous quadratic or spline specifications of overall engagement. In the statistical indirect association models, emotion-regulation variables were statistically involved in associations between moderate and high engagement profiles and mental health outcomes. However, the substantial overlap between cognitive reappraisal and expressive suppression limits strategy-specific interpretation of their mutually adjusted coefficients. Because all data were cross-sectional and self-reported, these findings describe association patterns rather than temporal or causal effects. They motivate future research on the emotional function, motivation, and quality of visual arts engagement rather than supporting conclusions about these unmeasured features in the present study.

## 1 Introduction

University students’ mental health is an important issue because this stage of life is often characterized by academic pressure, identity development, interpersonal adjustment, and multiple transition-related demands, and a substantial proportion of students experience psychological distress and role impairment during this period (Auerbach et al., 2018; Alonso et al., 2018). Visual arts engagement is relevant to this context because it may coincide with self-expression, mastery, aesthetic experience, and social connection. Self-determination theory provides one background lens for considering how activities that support autonomy, competence, and relatedness may relate to well-being, although these needs were not measured in the present study (Ryan and Deci, 2000; Krause et al., 2019). Research in arts and public health has likewise conceptualized arts participation as an everyday activity associated with psychological functioning, creative engagement, emotional expression, and social connection (Davies et al., 2012; Sonke et al., 2024). However, the relationship between visual arts engagement and mental health should not be assumed to be uniformly positive or linear, because engagement may have different psychological meanings depending on students’ motives, emotional states, and modes of participation.

An important question therefore remains: what does visual arts engagement actually look like among university students, especially those who are not art majors? Existing studies have more often focused on general arts engagement, art therapy contexts, or specific artistic populations, whereas comparatively less attention has been paid to the heterogeneity of visual arts engagement in the everyday lives of ordinary university students (Tomlinson et al., 2020; Sonke et al., 2024). As a result, it remains unclear whether non-art-major students can be meaningfully classified into different visual arts engagement profiles and whether these profiles are associated with differences in mental health. At the same time, even if visual arts engagement is related to mental health, the emotion-regulation patterns that accompany this relationship remain insufficiently described. Emotion regulation may be one theoretically relevant correlate, particularly because visual arts engagement involves the reinterpretation and expression of emotional experiences. Within Gross’s process model of emotion regulation, cognitive reappraisal and expressive suppression represent two theoretically distinct strategies that operate at different points in the emotion-generative sequence. Cognitive reappraisal involves changing the meaning of an emotional situation before the emotional response is fully generated, whereas expressive suppression involves inhibiting the outward expression of emotion after the emotional response has already been activated (Gross, 1998, 2015a; Gross and John, 2003). Visual arts engagement may therefore be associated with both strategies: it may involve the cognitive reframing of emotional experiences through imagery, symbolism, and aesthetic reflection, while also providing a nonverbal channel for emotional expression that may coincide with lower reliance on suppression. For this reason, the present study focuses on cognitive reappraisal and expressive suppression as two theoretically relevant emotion-regulation variables in the association between visual arts engagement and mental health. Accordingly, the present study aimed to identify latent profiles of visual arts engagement among Chinese non-art-major university students, examine whether these profiles are associated with differences in mental health, and describe the statistical involvement of emotion regulation in these cross-sectional associations.

## 2 Literature review

### 2.1 Non-art-major university students and visual arts participation

The university years represent an important transition from adolescence to early adulthood (Arnett, 2000). During this period, students are required to cope not only with coursework and academic pressure, but also with identity exploration, interpersonal adjustment, and future planning (Conley et al., 2014). As a result, mental health problems among university students are both prevalent and complex, involving not only negative forms of psychological distress such as anxiety, depression, and stress, but also lower well-being, reduced positive experience, and broader adjustment difficulties (Auerbach et al., 2018; Alonso et al., 2018; Conley et al., 2020). This makes it especially important to identify supportive resources embedded in everyday life that may facilitate psychological adjustment.

Visual arts engagement provides a meaningful perspective for addressing this issue. Unlike clinical or therapeutic contexts, visual arts engagement is often embedded in ordinary daily life and may include drawing, photography, appreciating artworks, browsing visual arts content, or participating in related classes and activities (Davies et al., 2012; Sonke et al., 2024). As a background theoretical perspective, self-determination theory suggests that activities experienced as autonomous, competence-supportive, or socially connected may coincide with more favorable adjustment (Ryan and Deci, 2000). The present study did not directly measure these psychological needs, however, and therefore does not test self-determination theory as an explanatory mechanism. For non-art-major university students, visual arts engagement is consequently treated here as an everyday form of participation rather than as professional training or artistic skill.

For non-art-major university students, the significance of visual arts engagement may be understood in at least two ways. First, such activities are often characterized by autonomy and self-expression, providing space for temporary disengagement from academic demands and for reflection on personal experience (Fancourt and Finn, 2019; Tomlinson et al., 2020). Second, visual arts engagement may foster positive emotion and psychological connectedness through aesthetic experience, creative involvement, and interaction with others (Tomlinson et al., 2020; Wang et al., 2020). From this perspective, visual arts engagement deserves closer attention in research on the mental health of non-art-major university students.

### 2.2 The concept of visual arts engagement and its heterogeneity among non-art-major university students

Visual arts engagement refers to the broader, multidimensional way individuals encounter and take part in visual arts activities in everyday life, including the forms and level of involvement (Davies et al., 2012). In the present study, participation refers to taking part in a specific visual arts activity, whereas engagement denotes the broader construct spanning active creation, receptive appreciation, digital engagement, and social/learning involvement. Frequency refers to how often such participation occurred during the specified assessment period and serves as the response metric used to operationalize participation within each dimension. Thus, the three terms are related but not interchangeable: participation describes the behavior, frequency quantifies its occurrence, and VAE represents the broader multidimensional pattern of participation assessed in the study.

This point is particularly important for non-art-major university students. Compared with art-major students, their visual arts engagement does not necessarily depend on systematic training or professional goals and is more likely to be embedded in ordinary study routines, leisure activities, and digital media use (Sonke et al., 2024). For this reason, their engagement may be characterized by greater everydayness, fragmentation, and diversity. Some students may engage mainly through appreciation and browsing, some may be more involved through creating, recording, or coursework, and others may show relatively high engagement across multiple dimensions (Bone et al., 2021). In other words, non-art-major university students are unlikely to be a fully homogeneous group with respect to visual arts engagement and may instead display different patterns and levels of engagement. This broader definition also suggests that relying only on mean scores may obscure important within-group differences.

Although existing research has increasingly emphasized diversity in arts engagement, empirical work has rarely focused on the internal heterogeneity of visual arts participation among non-art-major university students. Many studies treat arts engagement as a single continuous variable (Wang et al., 2020) or examine general arts engagement rather than visual arts specifically (Bone et al., 2021). The operationalization of arts engagement also varies by art form, mode of participation, and level or frequency of involvement (Davies et al., 2012; Sonke et al., 2024; Fan et al., 2024). In Chinese university students, Fan et al. (2024) distinguished creative and consumptive participation across visual, performing, and literary arts, illustrating why operational distinctions matter. Person-centered mixture approaches have likewise been applied to arts participation; Thornton et al. (2024), for example, used latent class analysis to identify patterns of adolescents’ arts, culture, and entertainment participation and examine later well-being. The present study therefore used latent profile analysis to examine how active creation, receptive appreciation, digital engagement, and social/learning involvement were jointly organized without assuming that qualitatively distinct profile shapes would emerge. Because level-based profiles may approximate an underlying continuum, continuous VAE models were also examined as a sensitivity analysis.

### 2.3 Previous studies on visual arts engagement and mental health

The present study adopts the dual-continua view of mental health, in which positive psychological functioning and psychological distress are related but distinct dimensions rather than opposite ends of a single continuum (Keyes, 2005; Westerhof and Keyes, 2010). Accordingly, mental health was operationalized using subjective well-being as a positive indicator and depression, anxiety, stress, and overall psychological distress as negative indicators. This framework allows visual arts engagement to show different associations with well-being and distress rather than assuming that one domain is simply the inverse of the other.

With regard to positive psychological functioning, arts engagement has often been associated with subjective well-being and flourishing (Cotter and Pawelski, 2022; Davies et al., 2016; Fan et al., 2024). Shim et al. (2021) found positive emotional, social, and sense-of-self changes across arts and humanities interventions, while Davies et al. (2016) reported an association between recreational arts engagement and mental well-being. Cotter and Pawelski (2022) similarly argued that art museums can support dimensions of human flourishing. More recently, Trupp et al. (2025) identified emotion regulation among the plausible mechanisms linking art viewing to well-being, and Rodriguez et al. (2024) conceptualized arts engagement as a potentially health-relevant behavior.

Evidence from Chinese university populations is consistent with this broader literature. Chen et al. (2022) found that receptive and digital arts engagement were associated with anxiety and resilience among isolated college students, whereas Fan et al. (2024) showed that creative and consumptive participation across art forms related differently to flourishing-related outcomes. These findings reinforce the value of specifying how arts engagement is operationalized rather than treating it as a unitary exposure.

With regard to negative psychological distress, an increasing body of literature suggests that arts engagement may also be associated with lower psychiatric symptoms and less emotional distress (Preacher and Hayes, 2008; Tein et al., 2013). In a recent high-level review, Fancourt et al. (2026) concluded that leisure arts activities, community arts programs, and creative arts therapies may be related to psychiatric symptomatology through multiple transdiagnostic processes. At the same time, this literature also suggests that the relationship between arts engagement and mental health is not always simple, linear, or uniform; different forms of arts engagement, different populations, and different outcome indicators may yield different patterns of association.

An important unresolved question concerns the psychological meaning and emotional function of different levels of visual arts engagement. Frequent engagement may reflect intrinsic interest, enjoyment, and meaningful self-expression for some students, but it may also function as emotion-focused coping, distraction, or distress-driven engagement for others. Art-making can influence mood differently depending on whether it is used for distraction, expression, or venting (Drake and Winner, 2012), while artistic activity can involve multiple emotion-regulation functions rather than a single uniformly adaptive process (Fancourt et al., 2019). People may also gravitate toward the arts during periods of elevated stress or disruption (Drake et al., 2024), underscoring the possibility of reverse directionality. A person-centered approach can describe subgroups with similar multivariate engagement patterns (Howard and Hoffman, 2018), while complementary continuous analyses can test whether those profiles add information beyond overall engagement level.

### 2.4 Cognitive reappraisal and expressive suppression as emotion-regulation correlates

Emotion regulation provides a useful theoretical lens for describing how visual arts engagement may be associated with mental health. According to Gross’s process and extended process models, emotions unfold through a sequence in which individuals attend to, appraise, and respond to emotionally relevant situations, with regulation possible at several stages (Gross, 1998, 2015a, 2015b). Cognitive reappraisal is an antecedent-focused strategy operating at cognitive change: it modifies the meaning of an emotional situation before the emotional response is fully generated. Expressive suppression is a response-focused strategy operating at response modulation: it inhibits or reduces outward emotional expression after the response has been activated (Gross and John, 2003). Thus, reappraisal primarily changes how an event is understood, whereas suppression changes how an already-aroused emotion is displayed.

Prior research has shown that cognitive reappraisal and expressive suppression have different implications for mental health. Cognitive reappraisal is generally regarded as a more adaptive strategy because it changes the interpretation of emotional events and is often associated with greater positive affect, higher well-being, better interpersonal functioning, and lower psychological distress. In contrast, expressive suppression is often associated with lower well-being, reduced emotional expression, poorer social functioning, and higher psychological symptoms, although its associations may vary across cultural and interpersonal contexts (Gross and John, 2003; Aldao et al., 2010; Lincoln et al., 2022). Given that the present study conceptualizes mental health as including both subjective well-being and psychological distress, distinguishing these two strategies is important for examining whether visual arts engagement is associated with similar or different emotion-regulation patterns across positive and negative mental health outcomes.

Visual arts engagement may be theoretically connected to both cognitive reappraisal and expressive suppression. Imagery, aesthetic reflection, symbolic transformation, and meaning-making may coincide with reinterpretation of emotional experience, whereas visual expression can provide a nonverbal channel for externalizing emotion. Research on artistic creative activities further indicates that engagement with the arts can serve multiple emotion-regulation functions. Fancourt et al. (2019), for example, identified distinct regulatory functions involving avoidance, approach, and self-development, illustrating that artistic activity should not be assumed to operate through a single emotional pathway. Art-making research has likewise emphasized expression, attentional redirection, and the organization of emotional experience (Gruber and Oepen, 2018). Recent review evidence further supports the relevance of emotion regulation to visual-art experiences. Trupp et al. (2025) identified emotion-regulatory processes among the plausible mechanisms through which viewing art may relate to well-being. This provides additional theoretical support for examining cognitive reappraisal and expressive suppression as emotion-regulation correlates of visual arts engagement. These links remain conceptual rather than causal in the present study, and periods of distress may themselves increase arts engagement (Drake et al., 2024).

Taken together, cognitive reappraisal and expressive suppression were selected as theoretically relevant emotion-regulation variables because they represent two distinct forms of emotion regulation within Gross’s process model and because both may be meaningfully connected to visual arts participation. Examining these two strategies allows the present study to move beyond asking whether visual arts engagement is associated with mental health and instead to describe whether cross-sectional association patterns also involve emotion-regulation variables.

### 2.5 Research gaps, research aims, and hypotheses

Although previous studies generally support associations between arts engagement and mental health, several gaps remain. First, much of the literature concerns general arts engagement rather than visual arts specifically, with comparatively little attention to non-art-major university students. Second, prior studies commonly use a single continuous engagement score, leaving the joint distribution of multiple engagement dimensions less well described; however, person-centered profiles should also be compared with continuous alternatives when profiles mainly differ by level. Third, cross-sectional associations involving emotion regulation remain insufficiently understood. The present study addresses these gaps while treating person-centered and variable-centered analyses as complementary rather than assuming that latent profiles necessarily provide superior prediction.

To address these gaps, the present study proceeded in three steps. First, latent profile analysis was used to identify potential profiles of visual arts engagement among Chinese non-art-major university students. Second, based on these profiles, the study examined whether different engagement profiles differed in mental health, with mental health being specified in terms of subjective well-being as the positive dimension and psychological distress as the negative dimension, consistent with the dual-continua perspective. Third, the study further tested whether emotion regulation showed statistical indirect associations with visual arts engagement profiles and mental health, in order to describe the statistical involvement of emotion regulation in these cross-sectional associations.

**Figure 1:**
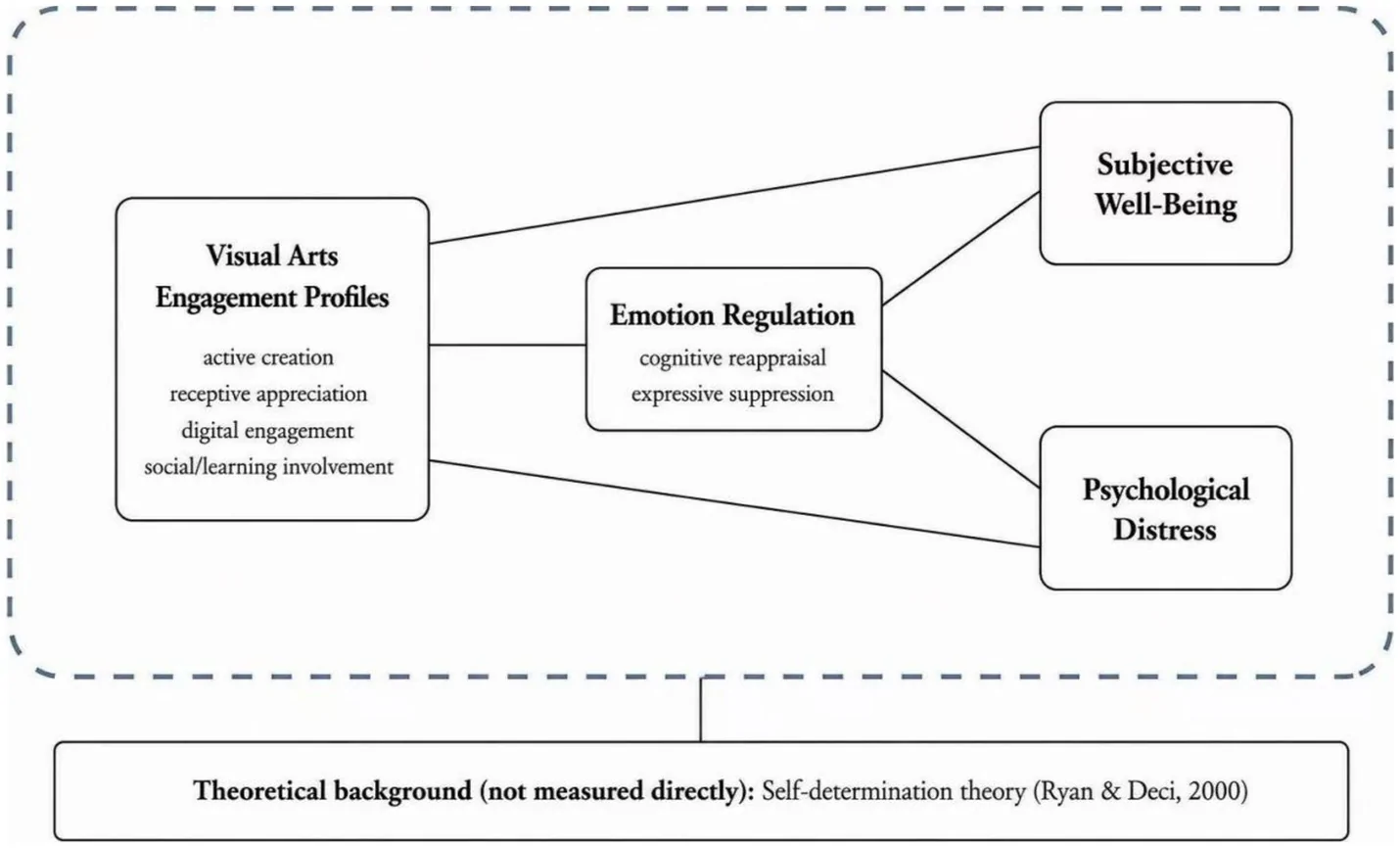
Conceptual association framework. Lines show cross-sectional associations only and imply no temporal or causal direction.

Based on these considerations, the following hypotheses were proposed, and the overall conceptual association framework examined in the present study is illustrated in Figure 1.

*H1*: Distinct latent profiles of visual arts engagement would emerge among non-art-major university students.

*H2*: Different latent profiles of visual arts engagement would differ significantly in mental health, including subjective well-being and psychological distress.

*H3*: Emotion regulation variables would be statistically involved in the cross-sectional associations between visual arts engagement profiles and mental health outcomes.

## 3 Materials and methods

### 3.1 Participants and sampling

This study employed a questionnaire-based cross-sectional survey design. Data were collected from May 1 to May 10, 2026. Participants were recruited from a university in northwestern China using convenience sampling, and the survey invitation was distributed through university-based student communication channels. The invitation described the study purpose, eligibility criteria, voluntary participation, anonymity, the availability of a small digital red-envelope monetary incentive, and an expected completion time of approximately 10 min. Participants were eligible if they were at least 18 years old and were not enrolled in art-related majors, such as fine arts, design, visual communication, photography, animation, crafts, art education, or media arts. No additional upper-age cutoff was specified beyond current university enrollment and the minimum-age criterion. The earliest retained de-identified survey export contained 586 records, all of which were included in the present analytic dataset. Because the exact number of students who received or viewed the invitation and any platform-level pre-export screening records were not retained, an exact response rate or larger initial response count could not be reconstructed reliably. The final sample was 51.71% female, 48.12% male, and 0.17% other, with a mean age of 20.97 years (SD = 2.71; range = 18–40 years). The demographic characteristics of the final analytic sample are presented in Table 1.

**Table 1:** Sample characteristics of the final analytic sample.

Characteristic	*n* (%) or M ± SD
Total sample	586
Female	303 (51.71%)
Male	282 (48.12%)
Other	1 (0.17%)
Age, years	20.97 ± 2.71 (18–40)

Values are presented as *n* (%) unless otherwise indicated. Age is presented as M ± SD, with the observed range shown in parentheses. M, mean; SD, standard deviation.

### 3.2 Procedure and data screening

Data were collected using an anonymous online questionnaire from May 1 to May 10, 2026. Participation was voluntary, a small digital red-envelope monetary incentive was offered, and the questionnaire was expected to require approximately 10 min to complete; per-response completion-time logs were not retained. At the beginning of the survey, participants were informed of the study purpose, voluntary participation, anonymity, and their right to withdraw without penalty, and only those who agreed to the informed-consent statement could proceed. Substantive scale items were required for submission. The retained de-identified dataset was checked for eligibility, completeness of required substantive items, exact duplicate response vectors, and broad response-pattern anomalies. No exact duplicate response vectors were identified. Because the survey was anonymous, IP addresses, device identifiers, and other direct identifiers were not retained and therefore could not be used for duplicate detection. One attention-check item instructed participants to select a specified response and was used as a descriptive quality indicator rather than as a sole exclusion criterion; it was not included in any scale score or statistical model. All 586 records in the earliest retained export were retained after these checks. Missing data on the substantive analysis variables were minimal because the main items were required, and no statistical imputation was applied.

#### 3.2.1 Ethics and informed consent

The study protocol was reviewed and approved by the relevant ethics authorities of the School of Fine Arts at the participating university in northwestern China. The study was conducted in accordance with the ethical principles of the Declaration of Helsinki and its subsequent amendments. All participants were adults aged 18 years or older and were informed of the study purpose, procedures, voluntary nature of participation, anonymity, data-confidentiality procedures, and their right to withdraw before providing informed consent.

### 3.3 Measures

#### 3.3.1 Translation and validation of survey instruments

Because the survey was administered in Chinese, the linguistic and psychometric appropriateness of all Chinese-language instruments was carefully considered. For standardized psychological instruments, previously published Chinese versions or official Chinese translation resources were used whenever available. Specifically, the Chinese version of the Emotion Regulation Questionnaire and the Chinese version of the WHO-5 Well-Being Index were adopted with reference to prior validation studies in Chinese-speaking or Chinese university student samples. For psychological distress, the official Chinese translation of the 21-item Depression Anxiety Stress Scales was used, and prior validation studies in Chinese samples were consulted to support its psychometric appropriateness. For the visual arts engagement item set, no single validated Chinese instrument was available that fully captured visual arts engagement among Chinese non-art-major university students. Therefore, the present study used an adapted exploratory item set developed with reference to established arts engagement frameworks. The items were translated, culturally adapted, and reviewed by bilingual researchers. A formal back-translation procedure was not applied because the items were not a direct translation of a single existing English-language scale. Instead, the review process focused on semantic clarity, conceptual consistency, cultural appropriateness, and relevance to everyday visual arts engagement among Chinese non-art-major university students. No separate formal pilot study was conducted before the main survey; therefore, the visual arts engagement measure should be regarded as an adapted exploratory inventory rather than a fully validated standalone scale. Current-sample reliability and confirmatory factor analysis were used to evaluate internal consistency and measurement structure, but these analyses do not replace independent pilot testing or external validation.

#### 3.3.2 Visual arts engagement (VAE)

VAE was assessed using a translated and adapted 14-item self-report inventory developed with reference to existing measures of arts engagement and arts participation (Davies et al., 2012; Sonke et al., 2024). Because no single validated Chinese instrument fully captured everyday visual arts engagement among Chinese non-art-major university students, the inventory was used as an exploratory measure for the present research context rather than as a fully validated standalone scale. Item development was guided by prior work that conceptualizes arts engagement in terms of art forms, activities, and levels of involvement, including active, receptive, digital, and socially embedded participation. The item pool was translated into Chinese and reviewed by bilingual researchers for semantic clarity, conceptual consistency, cultural appropriateness, and relevance to students’ everyday visual arts activities. Of the 14 items, 12 core items represented four dimensions: active creation (AC), receptive appreciation (RA), digital engagement (DE), and social/learning involvement (SI), with three items per dimension. The remaining two supplementary items captured broader contextual aspects of engagement and were not used as LPA indicators because they did not map directly onto the four prespecified dimensions. Participants reported how often they had engaged in each activity during the past 3 months on a 5-point scale ranging from 1 = never to 5 = very often. Accordingly, the present study operationalized the four VAE dimensions primarily through participation-frequency indicators. Dimension scores represented the average frequency of participation across the three activities within each dimension, while VAE was interpreted as the broader multidimensional engagement construct represented by these four dimensions. Cronbach’s *α* was 0.890, 0.872, 0.863, and 0.865 for AC, RA, DE, and SI, respectively, and 0.964 for the 12 core items. With ordinal items estimated using WLSMV, the four-factor CFA showed good approximate fit, *χ*^2^(48) = 172.35, CFI = 0.997, TLI = 0.996, RMSEA = 0.067, and SRMR = 0.013. Factor correlations were high (0.982–0.992), indicating substantial common-factor overlap and supporting cautious interpretation of the profile solution as predominantly level-based. The full wording of all 14 VAE items, including the 12 core items used to construct the four VAE dimensions and the two supplementary contextual items not included in the LPA, is provided in Supplementary Table S7.

#### 3.3.3 Emotion regulation (ER)

ER was measured using the Chinese version of the Emotion Regulation Questionnaire (ERQ), originally developed by Gross and John (2003). The Chinese ERQ has been examined in Chinese-speaking samples and has shown support for the intended two-factor structure, reliability, criterion validity, and measurement invariance across gender (Li and Wu, 2020). The ERQ assesses two habitual emotion-regulation strategies: cognitive reappraisal (CR) and expressive suppression (ES). The scale contains 10 items, including six CR items and four ES items, rated on a 7-point scale from 1 = strongly disagree to 7 = strongly agree. Higher scores indicate more frequent habitual use of the corresponding strategy, and subscale mean scores were calculated separately. In the present sample, Cronbach’s *α* was 0.948 for CR and 0.908 for ES. Using WLSMV with items treated as ordinal, the two-factor CFA showed good approximate fit, *χ*^2^(34) = 105.19, CFI = 0.998, TLI = 0.998, RMSEA = 0.060, and SRMR = 0.007. The latent correlation between CR and ES was high, a point considered in the later parallel statistical models.

#### 3.3.4 Subjective well-being (SWB)

SWB was assessed using the Chinese version of the World Health Organization-Five Well-Being Index (WHO-5), a brief self-report measure of positive mental well-being over the past 2 weeks (World Health Organization, 2024; Topp et al., 2015). The Chinese WHO-5 has been validated in mainland Chinese university student samples and has demonstrated good internal consistency, factorial validity, construct validity, and concurrent validity (Fung et al., 2022). The WHO-5 includes five positively worded items rated on a 6-point scale from 0 = at no time to 5 = all of the time. Higher scores indicate greater subjective well-being. Following standard scoring procedures, the five items were summed to produce a raw total score ranging from 0 to 25. The raw score, rather than the 0–100 transformed score, was used in all main analyses to avoid presenting duplicate versions of the same outcome. During the scoring audit, the exported WHO-5 item codes were found to use a 1–6 metric rather than the intended 0–5 response metric. Accordingly, 1 was subtracted from each exported WHO-5 response before the five corrected items were summed. All WHO-5 results reported in the revised manuscript are based on these corrected 0–5 item scores. In the present sample, Cronbach’s *α* was 0.913. Using WLSMV with items treated as ordinal, the one-factor CFA showed good approximate fit, *χ*^2^(5) = 14.87, CFI = 0.999, TLI = 0.997, RMSEA = 0.058, and SRMR = 0.010.

#### 3.3.5 Psychological distress (PD)

PD was assessed using the official Chinese version of the full 21-item Depression Anxiety Stress Scales (DASS-21), originally developed by Lovibond and Lovibond (1995). Prior research has supported the reliability and validity of the DASS-21 in Chinese samples, including Chinese university students (Moussa et al., 2001; Wang et al., 2016; Lu et al., 2018). The scale contains three 7-item subscales assessing depression, anxiety, and stress symptoms during the previous week. Participants indicated how much each statement applied to them on a 4-point response scale from 0 = did not apply to me at all to 3 = applied to me very much or most of the time. Subscale scores were calculated by summing the corresponding seven items and therefore ranged from 0 to 21; overall PD was calculated by summing all 21 items and ranged from 0 to 63. Scores were not multiplied by two because the present study treated the DASS-21 as a continuous research measure rather than converting scores to DASS-42-equivalent clinical severity categories. Before scale-score computation, the exported DASS-21 response codes were audited against the original questionnaire response anchors. For items exported on a 1–4 metric, 1 was subtracted from each response to restore the intended 0–3 scoring metric. No DASS-21 item was reverse-scored. Depression, anxiety, stress, and overall psychological-distress scores were subsequently recomputed from the corrected item-level data, and all analyses involving DASS-21 scores were rerun using these corrected scores. Cronbach’s *α* was 0.908, 0.856, 0.917, and 0.962 for depression, anxiety, stress, and overall PD, respectively. Using WLSMV with items treated as ordinal, the three-factor CFA showed good approximate fit, *χ*^2^(186) = 331.88, CFI = 0.997, TLI = 0.996, RMSEA = 0.037, and SRMR = 0.019. Item-level CFA results are reported in Supplementary Table S6; one anxiety item showed a weak loading in the present sample, so item-level measurement properties should be interpreted cautiously despite the acceptable overall three-factor fit and internal consistency.

### 3.4 Data analysis

The data analysis proceeded in four steps. First, descriptive statistics, observed ranges, distributional summaries, and internal consistency estimates were calculated for the major study variables. Primary mental-health outcomes were the WHO-5 raw score and DASS-21 overall psychological distress score; the three DASS-21 subscales were treated as related secondary outcomes. Exported WHO-5 and DASS-21 response codes were audited against their intended response anchors before scale scores were computed, with the exact recoding procedures reported in the respective Measures sections. Following correction, all WHO-5 and DASS-21 scale scores were recomputed, and all downstream analyses involving these variables, including descriptive statistics, reliability analyses, CFA, Welch’s ANOVA, Games–Howell comparisons, BCH analyses, and statistical indirect-association models, were rerun using the corrected scores.

Before the main analyses, confirmatory factor analyses evaluated the hypothesized four-factor VAE structure, two-factor ERQ structure, one-factor WHO-5 structure, and three-factor DASS-21 structure. Because the questionnaire indicators were ordered categorical responses, CFA models were estimated using robust weighted least squares (WLSMV), with the items treated as ordinal rather than continuous. Model fit was evaluated using the model *χ*^2^ statistic together with the comparative fit index (CFI), Tucker–Lewis index (TLI), root mean square error of approximation (RMSEA), and standardized root mean square residual (SRMR). The VAE model showed *χ*^2^(48) = 172.35, CFI = 0.997, TLI = 0.996, RMSEA = 0.067, and SRMR = 0.013; the ERQ model showed *χ*^2^(34) = 105.19, CFI = 0.998, TLI = 0.998, RMSEA = 0.060, and SRMR = 0.007; the WHO-5 model showed *χ*^2^(5) = 14.87, CFI = 0.999, TLI = 0.997, RMSEA = 0.058, and SRMR = 0.010; and the DASS-21 model showed *χ*^2^(186) = 331.88, CFI = 0.997, TLI = 0.996, RMSEA = 0.037, and SRMR = 0.019. Standardized loadings and latent-factor correlations were also inspected to evaluate measurement overlap and inform interpretation of the subsequent profile and regression analyses.

Second, latent profile analysis (LPA) was conducted in Mplus 8.3 to examine how heterogeneity in VAE was organized across AC, RA, DE, and SI. The four dimension scores were entered on their original 1–5 scales; they were not standardized, trimmed, or winsorized because their bounded response scales already limited extreme values. Models with one through five profiles were estimated using robust maximum likelihood (MLR). Under the prespecified LPA parameterization, class-specific indicator means were freely estimated, indicator variances were constrained to equality across profiles, and within-profile covariances were fixed to zero. Each model used 2,000 random starting-value sets, 500 of which were retained for final-stage optimization, with 20 initial-stage iterations. No nondefault convergence criterion was specified, and the Mplus default convergence criteria were used. The best log-likelihood was replicated for the three-, four-, and five-profile solutions. Model selection was based on the combined evidence from the log-likelihood, AIC, BIC, ABIC, entropy, VLMR, BLRT, class size, parsimony, and substantive interpretability rather than on any one criterion (Masyn, 2013; Nylund et al., 2007; Nylund-Gibson and Choi, 2018). Average posterior probabilities were examined in addition to entropy to assess classification precision. Most likely profile membership was used for Welch’s ANOVA, Games–Howell comparisons, and the descriptive statistical indirect association models; BCH sensitivity analyses were therefore conducted for the principal distal outcomes to evaluate whether profile differences persisted after adjusting for classification error. Because the retained profiles were predominantly level-based, an overall VAE score was also created by averaging the 12 core items and standardizing the resulting score. Linear, quadratic, and natural-spline models were estimated for the mental-health outcomes, after which profile membership was added to the nonlinear specifications. Nested-model *F* tests and changes in *R*^2^ were used to determine whether the profile representation contributed information beyond continuous VAE.

Third, Welch’s ANOVA was used to compare the VAE profiles on CR, ES, WHO-5, depression, anxiety, stress, and overall PD. Welch’s test was selected because profile sizes were unequal and homogeneity of variance could not be assumed; under these conditions it provides a more robust omnibus comparison than conventional one-way ANOVA (Welch, 1951; Delacre et al., 2019). When an omnibus effect was significant, Games–Howell *post hoc* tests were used to identify pairwise differences because this procedure accommodates unequal variances and group sizes (Games and Howell, 1976). Conventional *η*^2^, calculated as the between-group sum of squares divided by the total sum of squares, was reported descriptively to convey the magnitude of group membership differences, while statistical inference relied on Welch’s heteroscedasticity-robust test. Hedges’ *g* was reported for pairwise standardized mean differences, while Games–Howell mean differences with 95% confidence intervals were reported in Supplementary Table S1. The WHO-5 raw score and overall DASS-21 PD score were treated as the primary mental-health outcomes, with the DASS-21 subscales retained as related secondary outcomes. Benjamini–Hochberg false-discovery-rate corrections were applied as sensitivity checks across the related omnibus and pairwise tests.

Additional sensitivity analyses evaluated whether profile composition or measured background characteristics accounted for the main mental-health differences. Age was compared across profiles using Welch’s ANOVA, while gender, academic year, disciplinary area, and previous formal visual-arts training were compared using chi-square tests. Covariate-adjusted linear models for WHO-5 and overall PD included profile membership, age, gender, academic year, disciplinary area, previous formal visual-arts training, perceived family economic status, and available leisure time. HC3 heteroscedasticity-robust standard errors were used for inference. Extended sensitivity models additionally included recent academic stress and prior counseling or psychological treatment.

Finally, parallel statistical indirect association models were estimated in R 4.5.2 using the lavaan package (Rosseel, 2012). Because latent profile membership was multicategorical, three dummy variables represented the low, moderate, and high profiles, with the very low profile as the reference category (Hayes and Preacher, 2014). CR and ES were entered simultaneously as parallel statistical intermediary variables, and SWB and overall PD were modeled separately. The models reported all profile-to-ER paths, ER-to-outcome paths, direct profile-to-outcome associations, specific indirect associations through CR and ES, their combined indirect associations, total associations, standardized estimates, and *R*^2^. Indirect estimates were evaluated using 5,000 bootstrap resamples and percentile confidence intervals. Because CR and ES were strongly correlated, their zero-order correlation, variance-inflation factors, and simple-versus-mutually-adjusted regression coefficients were examined to assess multicollinearity and possible statistical suppression. The ordering of variables was theory-informed but not temporally established by the data. Accordingly, the models were interpreted as descriptive cross-sectional statistical association models rather than as evidence of temporal mediation or causal mechanisms.

## 4 Results

### 4.1 Model fit of the latent profile analysis

Before conducting the profile analyses, descriptive statistics and internal consistency estimates were examined for the principal study variables. Table 2 presents the full-sample means, standard deviations, observed ranges, and Cronbach’s *α* coefficients for the four VAE dimensions, overall VAE, emotion-regulation variables, and mental-health outcomes.

**Table 2:** Descriptive statistics and internal consistency of the principal study variables.

Variable	M	SD	Observed range	Cronbach’s *α*
Active creation (AC)	2.60	1.14	1–5	0.890
Receptive appreciation (RA)	2.65	1.12	1–5	0.872
Digital engagement (DE)	2.64	1.11	1–5	0.863
Social/learning involvement (SI)	2.64	1.11	1–5	0.865
Overall VAE	2.63	1.06	1–5	0.964
Cognitive reappraisal (CR)	4.58	1.51	1–7	0.948
Expressive suppression (ES)	4.55	1.50	1–7	0.908
WHO-5 well-being	14.94	6.39	0–25	0.913
Depression	8.47	5.58	0–21	0.908
Anxiety	8.64	5.10	0–21	0.856
Stress	8.64	5.68	0–21	0.917
Overall psychological distress	25.75	15.77	1–61	0.962

*N* = 586. VAE, visual arts engagement; WHO-5, World Health Organization-Five Well-Being Index. VAE, AC, RA, DE, and SI are reported as mean scores. CR and ES are ERQ subscale mean scores. WHO-5 is reported using its raw 0–25 score. Depression, anxiety, and stress are raw DASS-21 subscale scores ranging theoretically from 0 to 21, and overall psychological distress is the sum of the 21 DASS-21 items with a theoretical range of 0–63. Observed range refers to the minimum and maximum values present in the analytic sample.

An LPA was conducted using AC, RA, DE, and SI. One- through five-profile models were compared using LL, AIC, BIC, ABIC, entropy, VLMR, BLRT, and class proportions. The model fit indices are presented in Table 3.

**Table 3:** Fit indices for the one- to five-class latent profile models.

Model	LL	AIC	BIC	ABIC	Entropy	BLRT	VLMR	Class (%)
1-profile	−3588.942	7193.884	7228.870	7203.473	—	—	—	100
2-profile	−2721.192	5468.383	5525.236	5483.966	0.936	< 0.001	< 0.001	53.75/46.25
3-profile	−2310.164	4656.328	4735.047	4677.904	0.956	< 0.001	< 0.001	50.85/22.18/26.96
4-profile*	−2135.172	4316.343	4416.930	4343.913	0.943	< 0.001	< 0.001	22.01/29.69/26.11/22.18
5-profile	−2050.980	4157.960	4280.413	4191.523	0.945	< 0.001	< 0.001	21.84/29.86/25.77/14.51/8.02

LL, log-likelihood; AIC, Akaike information criterion; BIC, Bayesian information criterion; ABIC, sample-size adjusted BIC; BLRT, bootstrap likelihood ratio test; VLMR, Vuong–Lo–Mendell–Rubin likelihood ratio test. Entropy indicates classification accuracy. Class proportions are based on most likely profile membership. The four-profile solution was retained as a parsimonious person-centered representation, not because it minimized the information criteria.

AIC, BIC, and ABIC decreased continuously through the five-profile solution, and both VLMR and BLRT remained significant, so the four-profile solution was not selected because it had the best numerical fit. The 8.02% smallest profile in the five-profile model was also not treated as inherently too small or unusable. Instead, substantive comparison of the estimated profile means showed that the first three profiles of the five-profile solution were almost identical to the very low, low, and moderate profiles of the four-profile solution, with average engagement levels of approximately 1.34, 2.07, and 3.01. The main change introduced by the fifth profile was at the upper end of the continuum: the four-profile high-engagement group was divided into two parallel level-based profiles averaging approximately 3.97 and 4.65 across AC, RA, DE, and SI. Neither additional profile displayed a dimension-specific cross-over pattern, such as high creation combined with low appreciation or high digital engagement combined with low social involvement. Thus, moving from four to five profiles increased granularity at the high end of the same continuum rather than revealing a substantively new VAE configuration. The four-profile solution was therefore retained as a more parsimonious person-centered representation of the same underlying structure. Detailed class-specific means, classification characteristics, and indicator variances for the three-, four-, and five-profile solutions are provided in Supplementary Table S3.

The four-profile model showed entropy = 0.943 and relatively balanced modal proportions of 22.01, 29.69, 26.11, and 22.18%. Average posterior probabilities were 0.954, 0.957, 0.983, and 0.989 for the very low, low, moderate, and high VAE profiles, respectively, indicating high classification precision. The model-estimated average engagement levels were approximately 1.34, 2.07, 3.02, and 4.22. These results support interpretation of the retained profiles as empirically derived levels along a predominantly graded multicomponent engagement continuum.

### 4.2 Profile characteristics and labeling

The retained profiles primarily reflected increasing overall levels of VAE rather than qualitatively distinct shapes across indicators. They were therefore labeled very low VAE, low VAE, moderate VAE, and high VAE. This interpretation is consistent with the high correlations among the four VAE latent factors and with the parallel profile shapes across AC, RA, DE, and SI.

**Figure 2:**
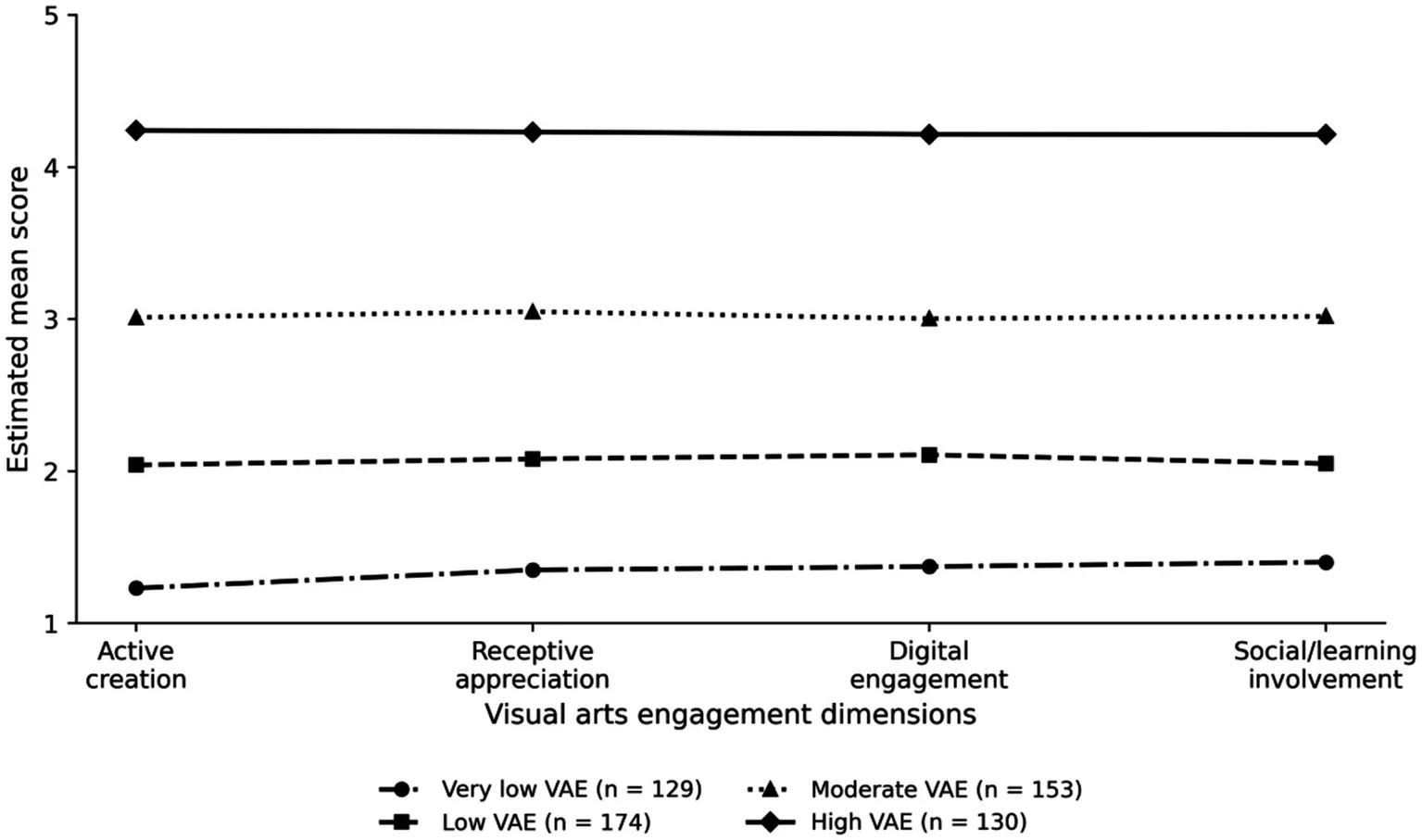
Profile plot of the four-profile latent profile solution. The figure displays model-estimated means for active creation, receptive appreciation, digital engagement, and social/learning involvement across the very low, low, moderate, and high VAE profiles.

Based on most likely class membership, profile sizes were 129 (22.01%), 174 (29.69%), 153 (26.11%), and 130 (22.18%), respectively. Continuous-model sensitivity analyses produced the same broad pattern. For WHO-5, the linear VAE model explained 15.0% of the variance, the quadratic model 15.1%, and the profile-only model 15.7%; adding profile membership to the quadratic model did not significantly improve explanatory power, Δ*R*^2^ = 0.007, *F*(3, 580) = 1.60, *p* = 0.189. For overall PD, the linear model explained 4.2% and the quadratic model 5.1%; a modest quadratic component was observed, B = 1.50, *p* = 0.022, but adding profiles was again nonsignificant, Δ*R*^2^ = 0.004, *F*(3, 580) = 0.85, *p* = 0.469. Natural-spline analyses yielded the same conclusion. Figure 2 presents the retained profile solution. Full continuous-model sensitivity results are provided in Supplementary Table S5.

### 4.3 Differences in ER and mental health outcomes across VAE profiles

After the four VAE profiles were identified, Welch’s ANOVA and Games–Howell *post hoc* comparisons examined group differences in ER and mental health. In the full sample, WHO-5 averaged 14.94 (SD = 6.39, observed range 0–25) and overall PD averaged 25.75 (SD = 15.77, observed range 1–61); floor and ceiling concentrations were low across the principal mental-health measures. As a descriptive benchmark, Fung et al. (2022) reported WHO-5 item means that sum to approximately 17.4–17.7 across two Chinese university samples, whereas Lu et al. (2018) reported DASS-21 depression, anxiety, and stress means of 2.17, 2.48, and 3.69, respectively. The present sample therefore showed somewhat lower well-being and higher distress than those comparison samples, although differences in sampling and study context preclude normative interpretation. Descriptive statistics, Welch results, and omnibus effect sizes are presented in Table 4.

**Table 4:** Descriptive statistics, Welch’s ANOVA, and omnibus effect sizes across the four VAE profiles.

Variable	Very low VAE M (SD)	Low VAE M (SD)	Moderate VAE M (SD)	High VAE M (SD)	Welch’s *F* (df1, df2)	*p*	*η* ^2^
CR	5.23 (1.35)	4.99 (1.34)	4.43 (1.40)	3.57 (1.47)	36.04 (3, 313.13)	<0.001	0.164
ES	5.22 (1.29)	4.91 (1.38)	4.39 (1.41)	3.62 (1.47)	33.19 (3, 314.63)	<0.001	0.150
WHO-5 raw score	17.63 (5.39)	16.74 (5.61)	14.10 (6.23)	10.86 (6.27)	35.27 (3, 313.74)	<0.001	0.157
Depression	7.67 (5.48)	7.41 (4.98)	8.52 (5.56)	10.63 (5.90)	9.20 (3, 310.45)	<0.001	0.049
Anxiety	8.17 (4.95)	7.66 (4.55)	8.27 (4.92)	10.84 (5.54)	9.93 (3, 310.09)	<0.001	0.055
Stress	7.84 (5.73)	7.55 (5.02)	8.74 (5.47)	10.80 (6.15)	8.80 (3, 309.12)	<0.001	0.048
Overall PD	23.67 (15.60)	22.62 (13.86)	25.53 (15.34)	32.27 (17.04)	9.85 (3, 309.36)	<0.001	0.054

Values are presented as M (SD). VAE, visual arts engagement; CR, cognitive reappraisal; ES, expressive suppression; WHO-5, World Health Organization-Five Well-Being Index; PD, psychological distress. WHO-5 is reported using its raw 0–25 score. *η*^2^ is the conventional descriptive omnibus effect-size index, calculated as SS_between/SS_total; inferential tests are based on Welch’s heteroscedasticity-robust *F* statistic. All seven omnibus profile effects remained significant after Benjamini–Hochberg false-discovery-rate correction.

Welch analyses indicated significant profile differences for CR, ES, WHO-5, depression, anxiety, stress, and overall PD. All seven omnibus profile effects remained significant after Benjamini–Hochberg false-discovery-rate correction. Omnibus *η*^2^ values ranged from 0.048 to 0.164. CR, ES, and WHO-5 generally decreased from the very low to the high VAE profile, whereas depression, anxiety, stress, and overall PD were highest in the high VAE profile. To clarify the specific pairwise differences underlying these omnibus effects, Table 5 summarizes the Games–Howell comparison patterns together with the corresponding Hedges’ *g* ranges for significant contrasts.

**Table 5:** Summary of Games–Howell *post hoc* comparisons and pairwise effect sizes across the four VAE profiles.

Variable	Nonsignificant comparison(s)	Significant comparison pattern	Hedges’ *g* range for significant comparisons
CR	Very low VAE vs. low VAE	Very low VAE > moderate VAE; very low VAE > high VAE; low VAE > moderate VAE; low VAE > high VAE; moderate VAE > high VAE	0.406 to 1.172
ES	Very low VAE vs. low VAE	Very low VAE > moderate VAE; very low VAE > high VAE; low VAE > moderate VAE; low VAE > high VAE; moderate VAE > high VAE	0.373 to 1.155
WHO-5 raw score	Very low VAE vs. low VAE	Very low VAE > moderate VAE; very low VAE > high VAE; low VAE > moderate VAE; low VAE > high VAE; moderate VAE > high VAE	0.444 to 1.154
Depression	Very low VAE vs. low VAE; very low VAE vs. moderate VAE; low VAE vs. moderate VAE	High VAE > very low VAE; high VAE > low VAE; high VAE > moderate VAE	0.369 to 0.595
Anxiety	Very low VAE vs. low VAE; very low VAE vs. moderate VAE; low VAE vs. moderate VAE	High VAE > very low VAE; high VAE > low VAE; high VAE > moderate VAE	0.490 to 0.634
Stress	Very low VAE vs. low VAE; very low VAE vs. moderate VAE; low VAE vs. moderate VAE	High VAE > very low VAE; high VAE > low VAE; high VAE > moderate VAE	0.355 to 0.587
Overall PD	Very low VAE vs. low VAE; very low VAE vs. moderate VAE; low VAE vs. moderate VAE	High VAE > very low VAE; high VAE > low VAE; high VAE > moderate VAE	0.416 to 0.629

“>” indicates that the group on the left scored significantly higher than the group on the right. Hedges’ *g* is reported in absolute magnitude for significant comparisons.

Games–Howell *post hoc* comparisons further clarified these group differences. For CR, ES, and WHO-5, the very low and low VAE profiles did not differ significantly from one another, whereas both generally scored significantly higher than the moderate and high profiles. The moderate profile also scored significantly higher than the high profile on all three variables. Hedges’ *g* values for significant comparisons ranged from 0.373 to 1.172, with the largest standardized differences observed in comparisons involving the high VAE profile. The overall pattern therefore remained graded for emotion regulation and subjective well-being, but the largest separation occurred at the upper end of engagement.

For depression, anxiety, stress, and overall PD, differences were concentrated in the high VAE profile. The very low, low, and moderate profiles did not differ significantly from one another on these distress indicators, whereas the high profile scored significantly higher than all three lower-engagement profiles. Absolute Hedges’ g values for significant comparisons ranged from 0.355 to 0.634. Thus, the high VAE profile showed the least favorable mental-health pattern in this sample, with lower subjective well-being and higher psychological distress.

To evaluate whether these distal-outcome differences were sensitive to classification uncertainty, BCH analyses were rerun using the corrected WHO-5 and DASS-21 scores. The BCH-adjusted means closely reproduced the modal-class pattern. WHO-5 means declined from 17.672 in the very low profile to 16.720, 14.069, and 10.827 in the low, moderate, and high profiles, respectively, and the overall BCH test was significant, *χ*^2^(3) = 107.030, *p* < 0.001. Overall PD means were 23.728, 22.558, 25.564, and 32.341, with a significant overall test, *χ*^2^(3) = 29.893, *p* < 0.001. The overall BCH tests were also significant for CR, *χ*^2^(3) = 109.392, ES, *χ*^2^(3) = 100.712, depression, *χ*^2^(3) = 27.924, anxiety, *χ*^2^(3) = 30.146, and stress, *χ*^2^(3) = 26.702, all *p* < 0.001. In each distress outcome, the high VAE profile retained the highest adjusted mean, while the high profile retained the lowest WHO-5 mean. These analyses therefore indicate that the central distal-outcome pattern was not an artifact of assigning students to their single most likely profile. Corrected BCH results are reported in Supplementary Table S2.

Additional analyses examined whether the four VAE profiles differed in available background characteristics. No significant profile differences were found for age, Welch’s *F*(3, 319.36) = 1.31, *p* = 0.272, gender, *χ*^2^(6) = 3.22, *p* = 0.780, academic year, *χ*^2^(15) = 13.60, *p* = 0.556, disciplinary area, *χ*^2^(21) = 30.57, *p* = 0.081, or previous formal visual-arts training, *χ*^2^(12) = 10.54, *p* = 0.569. In covariate-adjusted models using HC3 robust inference, profile membership remained jointly associated with WHO-5, *F*(3, 555) = 37.82, *p* < 0.001, and overall psychological distress, *F*(3, 555) = 8.40, *p* < 0.001. Relative to the very low VAE profile, the high VAE profile remained associated with lower WHO-5 scores, B = −7.06, 95% CI [−8.50, −5.62], *p* < 0.001, and higher overall PD, B = 8.67, 95% CI [4.47, 12.86], *p* < 0.001. Extended models additionally including recent academic stress and prior counseling or psychological treatment yielded the same substantive pattern.

### 4.4 Statistical indirect associations involving ER

Parallel statistical indirect association models were then used to describe the statistical involvement of ER in the associations between VAE profiles and psychological outcomes. The very low VAE profile served as the reference group, and indirect associations were estimated with 5,000 bootstrap resamples. Before interpreting the parallel coefficients, we examined the relationship between the two ER variables. CR and ES were strongly correlated, r = 0.910, 95% CI [0.895, 0.923], and their variance-inflation factors were approximately 5.9 when entered together. The full zero-order correlation matrix is provided in Supplementary Table S4. These diagnostics indicate substantial shared variance even though they do not by themselves make model estimation impossible. The mutually adjusted ER coefficients, especially the ES coefficient, were therefore interpreted cautiously and compared with the corresponding simple regressions to evaluate possible statistical suppression. In the SWB model, the profile variables explained 16.4% of the variance in CR (*R*^2^ = 0.164) and 15.0% in ES (*R*^2^ = 0.150), while the full model explained 72.0% of the variance in WHO-5 (*R*^2^ = 0.720).

**Figure 3:**
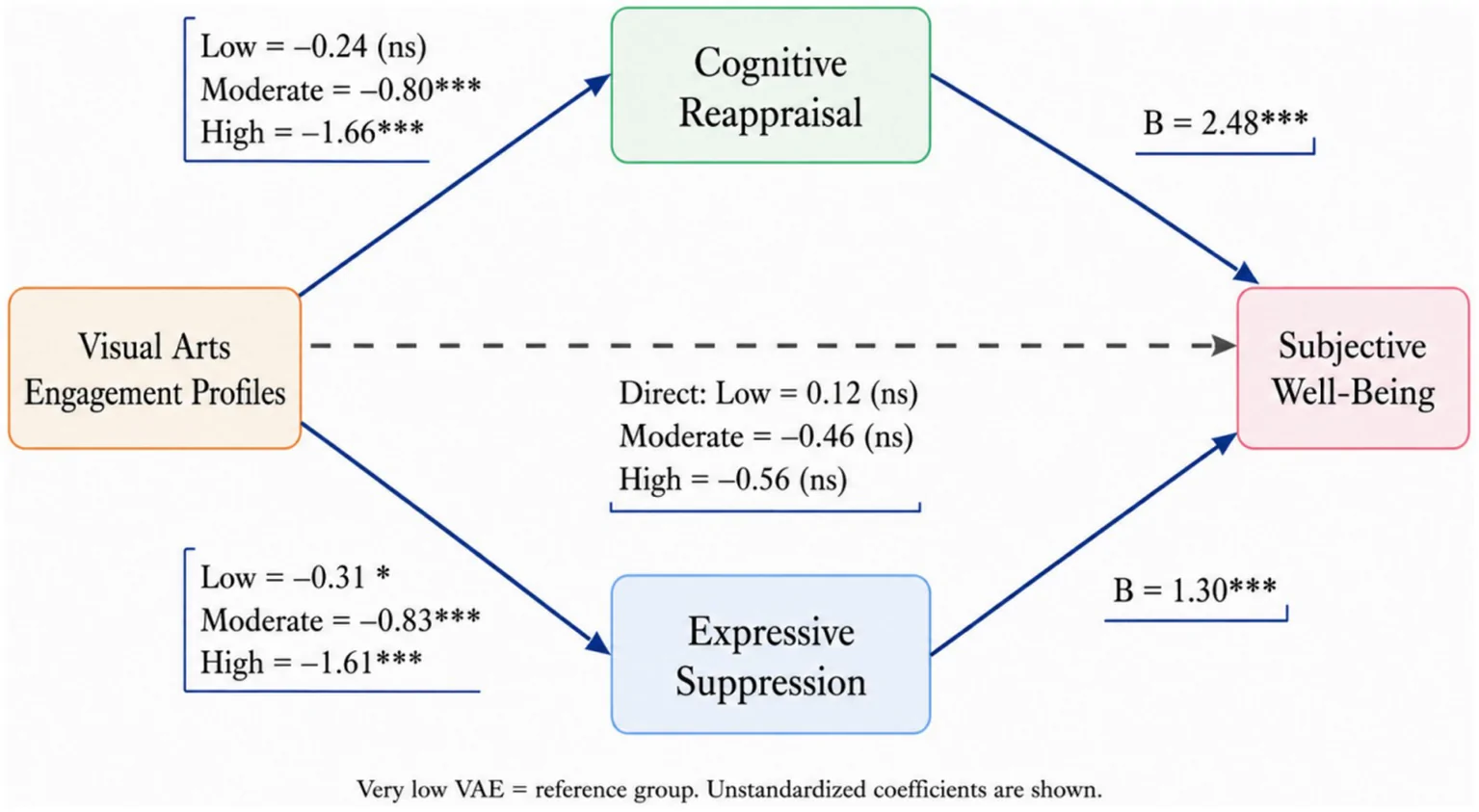
Cross-sectional statistical association coefficient diagram involving ER and SWB. The very low VAE profile served as the reference group. CR and ES were parallel statistical intermediary variables and SWB was the WHO-5 raw score. Lines depict regression coefficients in a cross-sectional statistical model and should not be interpreted as temporal or causal. Unstandardized coefficients are shown. ns = nonsignificant. * *p* < 0.05. ** *p* < 0.01. *** *p* < 0.001.

In the SWB model, CR (B = 2.477, *p* < 0.001) and ES (B = 1.305, *p* < 0.001) were positively associated with WHO-5 scores. Relative to the very low profile, moderate and high VAE were associated with lower CR and ES; the low-profile coefficient was nonsignificant for CR and small for ES. Specific indirect associations through CR were significant for the moderate profile (B = −1.978, 95% CI [−2.913, −1.125]) and high profile (B = −4.110, 95% CI [−5.383, −2.962]); corresponding indirect associations through ES were also significant (moderate: B = −1.088, 95% CI [−1.778, −0.535]; high: B = −2.095, 95% CI [−3.126, −1.187]). Combined indirect associations were −3.066 and −6.206 for the moderate and high profiles, respectively, whereas the low-profile combined indirect association was not significant. Direct profile-to-SWB coefficients were nonsignificant. Table 6 and Figure 3 summarize the model.

**Table 6:** Parallel statistical indirect association model involving ER and SWB.

Association/path	B	SE	95% CI	*p*	*β*
Low VAE → CR	−0.243	0.157	[−0.557, 0.063]	0.123	−0.073
Moderate VAE → CR	−0.798	0.164	[−1.126, −0.480]	<0.001	−0.232
High VAE → CR	−1.659	0.177	[−2.013, −1.315]	<0.001	−0.456
Low VAE → ES	−0.313	0.155	[−0.619, −0.003]	0.044	−0.095
Moderate VAE → ES	−0.834	0.161	[−1.146, −0.514]	<0.001	−0.244
High VAE → ES	−1.606	0.174	[−1.943, −1.254]	<0.001	−0.444
CR → SWB	2.477	0.255	[1.968, 2.965]	<0.001	0.686
ES → SWB	1.305	0.269	[0.777, 1.828]	<0.001	0.360
Direct: low vs. very low	0.118	0.311	[−0.493, 0.735]	0.704	0.010
Direct: moderate vs. very low	−0.458	0.387	[−1.226, 0.297]	0.237	−0.037
Direct: high vs. very low	−0.561	0.363	[−1.271, 0.137]	0.122	−0.043
Indirect via CR: low	−0.602	0.395	[−1.395, 0.160]	0.127	−0.050
Indirect via CR: moderate	−1.978	0.455	[−2.913, −1.125]	<0.001	−0.159
Indirect via CR: high	−4.110	0.611	[−5.383, −2.962]	<0.001	−0.313
Indirect via ES: low	−0.408	0.227	[−0.910, −0.004]	0.072	−0.034
Indirect via ES: moderate	−1.088	0.320	[−1.778, −0.535]	<0.001	−0.088
Indirect via ES: high	−2.095	0.499	[−3.126, −1.187]	<0.001	−0.160
Combined indirect: low	−1.010	0.580	[−2.170, 0.131]	0.082	−0.085
Combined indirect: moderate	−3.066	0.611	[−4.262, −1.887]	<0.001	−0.247
Combined indirect: high	−6.206	0.673	[−7.539, −4.932]	<0.001	−0.473
Total: low vs. very low	−0.892	0.643	[−2.137, 0.395]	0.165	−0.075
Total: moderate vs. very low	−3.523	0.692	[−4.875, −2.204]	<0.001	−0.284
Total: high vs. very low	−6.766	0.732	[−8.195, −5.294]	<0.001	−0.516

**Figure 4:**
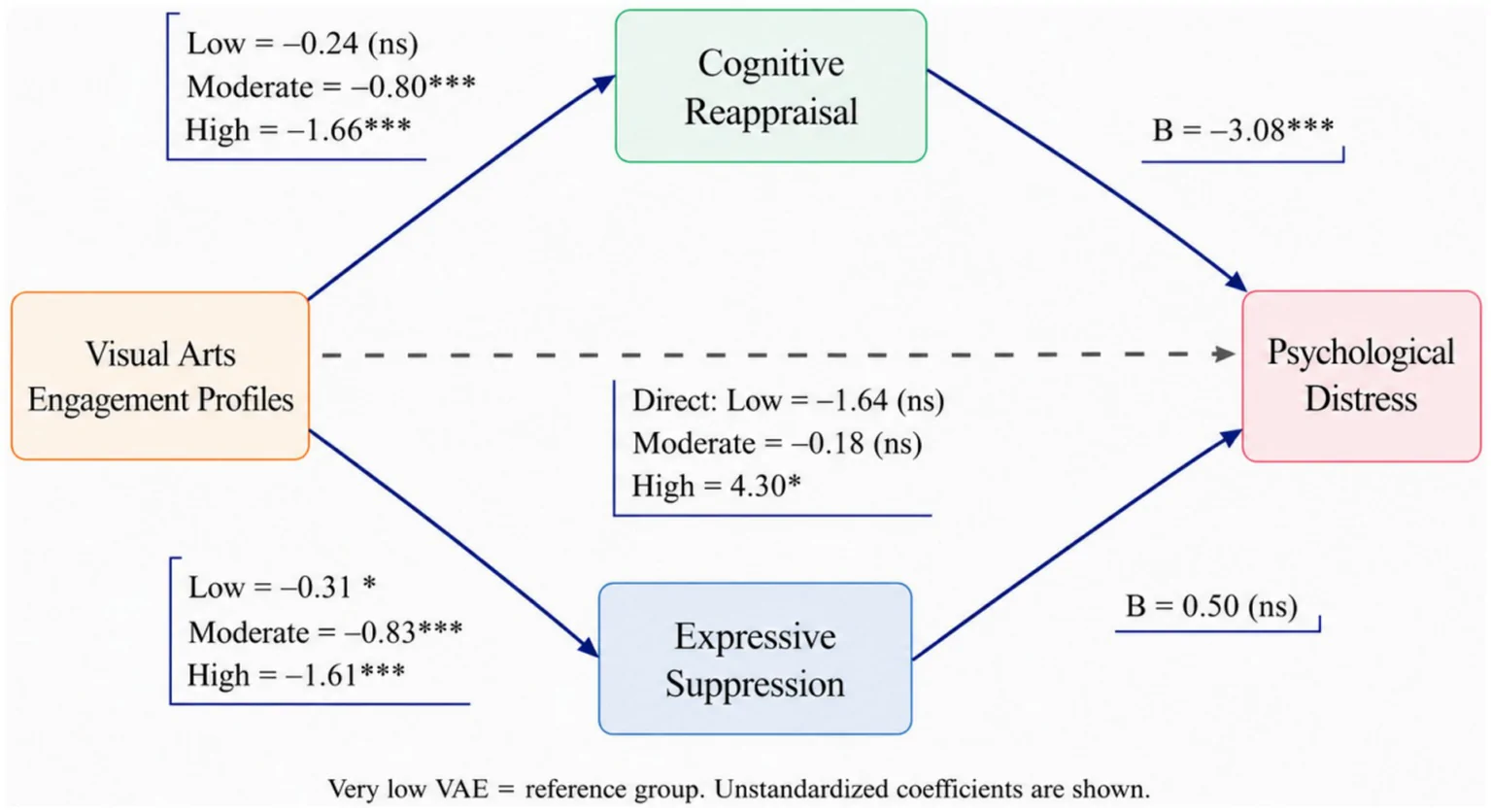
Cross-sectional statistical association coefficient diagram involving ER and overall PD. The very low VAE profile served as the reference group. CR and ES were parallel statistical intermediary variables and overall PD was the DASS-21 total score. Lines depict regression coefficients in a cross-sectional statistical model and should not be interpreted as temporal or causal. Unstandardized coefficients are shown. ns = nonsignificant. *p < 0.05. **p < 0.01. ***p < 0.001.

In the overall PD model, CR was negatively associated with distress (B = −3.076, *p* < 0.001), whereas the mutually adjusted ES coefficient was nonsignificant (B = 0.505, *p* = 0.585). The simple ES–PD association was negative, but its sign changed after simultaneous adjustment for CR, consistent with substantial shared variance and possible statistical suppression. Specific indirect associations through CR were significant for the moderate profile (B = 2.456, 95% CI [0.824, 4.406]) and high profile (B = 5.105, 95% CI [1.908, 8.495]); all specific ES indirect associations included zero. The combined indirect association was significant for the moderate profile (B = 2.035, 95% CI [0.960, 3.436]) and high profile (B = 4.295, 95% CI [2.517, 6.195]). The adjusted direct coefficient remained significant only for the high profile (B = 4.300, *p* = 0.036), and its total association was B = 8.595, 95% CI [4.624, 12.500]. Table 7 and Figure 4 summarize the model. The model explained *R*^2^ = 0.126 of overall PD.

**Table 7:** Parallel statistical indirect association model involving ER and overall PD.

Association/path	B	SE	95% CI	*p*	*β*
Low VAE → CR	−0.243	0.157	[−0.557, 0.063]	0.123	−0.073
Moderate VAE → CR	−0.798	0.164	[−1.126, −0.480]	<0.001	−0.232
High VAE → CR	−1.659	0.177	[−2.013, −1.315]	<0.001	−0.456
Low VAE → ES	−0.313	0.155	[−0.619, −0.003]	0.044	−0.095
Moderate VAE → ES	−0.834	0.161	[−1.146, −0.514]	<0.001	−0.244
High VAE → ES	−1.606	0.174	[−1.943, −1.254]	<0.001	−0.444
CR → overall PD	−3.076	0.928	[−4.844, −1.179]	<0.001	−0.292
ES → overall PD	0.505	0.925	[−1.402, 2.276]	0.585	0.048
Direct: low vs. very low	−1.644	1.685	[−4.939, 1.735]	0.329	−0.047
Direct: moderate vs. very low	−0.180	1.851	[−3.765, 3.415]	0.922	−0.005
Direct: high vs. very low	4.300	2.051	[0.220, 8.380]	0.036	0.112
Indirect via CR: low	0.748	0.562	[−0.189, 2.044]	0.183	0.021
Indirect via CR: moderate	2.456	0.924	[0.824, 4.406]	0.008	0.068
Indirect via CR: high	5.105	1.650	[1.908, 8.495]	0.002	0.133
Indirect via ES: low	−0.158	0.333	[−0.905, 0.475]	0.635	−0.005
Indirect via ES: moderate	−0.421	0.786	[−1.966, 1.197]	0.593	−0.012
Indirect via ES: high	−0.810	1.495	[−3.733, 2.249]	0.588	−0.021
Combined indirect: low	0.590	0.465	[−0.261, 1.583]	0.204	0.017
Combined indirect: moderate	2.035	0.636	[0.960, 3.436]	0.001	0.056
Combined indirect: high	4.295	0.941	[2.517, 6.195]	<0.001	0.112
Total: low vs. very low	−1.054	1.695	[−4.404, 2.354]	0.534	−0.030
Total: moderate vs. very low	1.855	1.820	[−1.715, 5.438]	0.308	0.051
Total: high vs. very low	8.595	1.979	[4.624, 12.500]	<0.001	0.224

## 5 Discussion

### 5.1 Summary of the main findings

The present study examined visual arts engagement among Chinese non-art-major university students using both person-centered and variable-centered analyses. Four empirically derived level-based profiles were retained as a parsimonious description of the sample, but continuous sensitivity models showed that profile membership did not add significant explanatory value beyond continuous nonlinear VAE specifications. The high VAE profile nevertheless showed the lowest subjective well-being and the highest psychological distress in the cross-sectional data. Emotion-regulation variables were statistically involved in these associations, although the strong overlap between cognitive reappraisal and expressive suppression limits strategy-specific interpretation. Higher engagement was therefore not linked to better mental health in this sample, but the design cannot determine why this pattern occurred.

### 5.2 Latent profiles of visual arts engagement among non-art-major university students

The LPA identified very low, low, moderate, and high VAE profiles that primarily differed in overall level rather than in qualitatively distinct configurations. Comparison with the five-profile solution showed that the additional profile mainly split the upper end of the same continuum into two parallel levels, while the first three profiles remained nearly unchanged. Continuous linear, quadratic, and spline models further showed that the retained profiles did not provide significant incremental explanatory value for WHO-5 or overall PD beyond continuous VAE. The LPA should therefore be understood as a person-centered descriptive representation of a graded multicomponent continuum rather than as a superior predictive model.

### 5.3 Visual arts engagement and mental health: a complex association

A central cross-sectional pattern was that students in the high-engagement profile reported lower WHO-5 well-being and higher depression, anxiety, stress, and overall psychological distress than students in the lower-engagement profiles. This pattern should not be interpreted as evidence that visual arts engagement is harmful.

One plausible explanation is reverse or bidirectional association. Students experiencing greater emotional difficulty may turn to visual arts for expression, distraction, self-soothing, identity work, or temporary relief from academic and interpersonal stress. In that case, high engagement would partly reflect coping with pre-existing distress rather than causing poorer mental health. This interpretation is consistent with longitudinal evidence that mental health can shape arts engagement as well as vice versa (Mak et al., 2024) and with evidence that people may gravitate toward artistic activity during stressful periods (Drake et al., 2024). A second possibility concerns the function of engagement. Artistic activity can serve multiple regulatory purposes rather than a single uniformly beneficial one (Fancourt et al., 2019). For example, art-making used for distraction may improve mood more effectively than art-making centered on venting negative emotion (Drake and Winner, 2012). The present study did not measure motivation, emotional function, or quality of participation, so these explanations remain hypotheses for future research rather than conclusions supported directly by the current data.

The context of engagement may also matter. Contemporary visual arts participation often includes fragmented digital activities such as browsing images, design content, or online artworks. These activities may provide aesthetic pleasure but can also coincide with passive consumption, social comparison, or self-criticism (Appel et al., 2016; Verduyn et al., 2015). The present results therefore support a narrower claim: in this cross-sectional sample, greater frequency of multicomponent VAE was not consistently associated with more favorable mental-health indicators. Future work should test which motives, contexts, and forms of engagement account for this pattern.

### 5.4 Emotion regulation patterns in the statistical association models

Emotion regulation was associated with both VAE profiles and mental-health outcomes. Moderate and high VAE profiles reported lower CR and ES than the lower-engagement profiles. In the parallel statistical models, the CR-specific indirect estimates were numerically larger and more consistent than the ES-specific estimates. However, CR and ES were very strongly correlated, and their mutually adjusted coefficients showed substantial multicollinearity and evidence of statistical suppression. The relative magnitudes of the CR- and ES-specific coefficients should therefore not be interpreted as evidence that one strategy played a uniquely stronger psychological role than the other. More conservatively, the findings indicate that shared variation in the measured emotion-regulation tendencies was statistically involved in the observed associations between VAE profiles and mental health.

The CR-related coefficient pattern is broadly compatible with prior theory on cognitive reappraisal, which conceptualizes reappraisal as an antecedent-focused strategy that changes how an emotional situation is interpreted before the response is fully generated (Gross, 2015a; Gross and John, 2003). However, its strategy-specific meaning is difficult to isolate in the present data because of the very strong overlap between CR and ES. Visual arts activities can provide opportunities for symbolic processing, attentional redirection, aesthetic distance, and meaning-making, all of which may conceptually overlap with reframing emotional experience (Ren et al., 2026). However, the present data do not show that visual arts engagement actually trains or improves reappraisal. In fact, students with higher VAE reported lower habitual reappraisal. The finding is therefore better understood as a cross-sectional covariation pattern involving emotion-regulation tendencies, rather than as evidence that a specific strategy or arts-based regulatory mechanism operated in this sample.

Further caution is warranted when interpreting the mutually adjusted ES coefficients. CR and ES were highly correlated (*r* = 0.910), and their VIFs were approximately 5.9 in the parallel models. Moreover, ES was negatively associated with PD when modeled alone but changed direction and became nonsignificant after CR was entered simultaneously. This sign change indicates substantial shared variance and possible statistical suppression. Accordingly, the positive mutually adjusted ES coefficient for SWB should not be interpreted as evidence that suppression is generally adaptive.

Cultural context may still be relevant to how self-reported suppression is understood. In Chinese and broader East Asian contexts, emotional restraint can sometimes be linked to self-control, relational appropriateness, ideal affect, and interpersonal harmony (Butler et al., 2007; Markus and Kitayama, 1991; Tsai, 2007; Wei et al., 2013). At the same time, the ERQ does not distinguish rigid suppression from context-sensitive restraint, strategic concealment, or flexible regulation of emotional display. Cultural interpretation therefore cannot resolve the statistical overlap between CR and ES and should remain secondary to the observed multicollinearity.

Measurement boundaries also matter. The ERQ captures habitual self-reported tendencies rather than momentary regulatory choices, and both ER and mental-health outcomes were assessed using self-report instruments. Shared method variance, response style, and unmeasured third variables may therefore contribute to the associations. The ES results are best treated as model-dependent coefficients requiring replication rather than as evidence for a beneficial role of expressive suppression.

More broadly, the indirect association models do not establish a temporal sequence from VAE to emotion regulation to mental health. Students with lower well-being or higher distress may engage more frequently in visual arts as a coping strategy, and distress may influence both ER strategy use and engagement. The models therefore describe how these variables covary in the present sample rather than identifying an explanatory process.

These limitations are especially important because profile membership itself largely reflected an underlying engagement continuum. The statistical indirect findings should therefore be read alongside the continuous sensitivity analyses and the strong overlap between CR and ES, rather than as evidence of discrete psychological types or causal pathways.

## 6 Practical implications

The findings motivate tentative hypotheses for university practice rather than tested implementation recommendations. Future programs could examine whether students’ motives, emotional needs, and experiences during participation help distinguish supportive engagement from distress-driven coping. The rationale for testing low-pressure and non-evaluative formats comes from the observed coexistence of high VAE with lower well-being and higher psychological distress in the present sample. If some highly engaged students are participating partly in response to distress, additional performance demands, artistic comparison, or compulsory emotional disclosure could plausibly add pressure rather than provide support. Accordingly, intervention studies could test whether culturally appropriate guided reflection, artifact-centered discussion, symbolic expression, aesthetic distance, optional sharing, and low-pressure non-evaluative settings improve emotion regulation or well-being. These possibilities remain hypotheses to be evaluated prospectively rather than direct implications established by the present cross-sectional data.

In Chinese university settings, reflective components may be most acceptable when they are low-pressure, indirect, and embedded in familiar educational or campus contexts rather than framed as mandatory clinical disclosure. General education art courses, exhibitions, student clubs, or counseling-center workshops could use brief prompts about the meaning of an artwork, the situation or feeling it represents, and alternative interpretations, while emphasizing respectful listening and meaning-making rather than artistic evaluation. Their effectiveness, acceptability, and possible unintended effects should be tested experimentally and through process evaluation.

Because the present study measured general visual arts engagement rather than any specific traditional practice, examples such as calligraphy, ink painting, paper cutting, or Chinese shadow puppetry remain exploratory contexts for future research rather than recommended treatments.

## 7 Limitations and directions for future research

Several limitations should be acknowledged. First, because the study was cross-sectional, the temporal direction of the association between VAE and mental health cannot be established. Poorer mental health may increase subsequent engagement with visual arts as a form of coping, just as engagement may potentially relate to later psychological functioning. Longitudinal cross-lagged and experimental designs are needed to distinguish these possibilities. The indirect association models should therefore not be interpreted as establishing temporal mediation or an explanatory process. Future research should use longitudinal panels, experience-sampling methods, cross-lagged or dynamic models, and experimental designs to examine directionality. For example, a randomized study could compare a structured reappraisal-oriented visual arts condition, an unguided expressive condition, and a non-arts control condition while measuring both short-term regulatory responses and later well-being. Such designs would provide substantially stronger evidence about whether particular forms of visual arts engagement alter emotion-regulation processes or whether engagement primarily reflects pre-existing psychological states.

Second, the VAE item set was an adapted exploratory inventory rather than a fully validated standalone Chinese scale. Although current-sample reliability and ordinal CFA supported its use, no separate pilot study or formal back-translation was conducted, and the four VAE factors were extremely highly correlated. This common-factor overlap is consistent with the largely level-based profile structure and limits claims about sharply differentiated dimensions. Future work should use cognitive interviewing, independent validation, test–retest assessment, and broader item pools that may capture more configuration-specific engagement.

Third, BCH sensitivity analyses adjusted distal outcome comparisons for classification error, but the statistical indirect association models still used most likely profile membership. Classification precision was high, yet these models do not fully propagate latent-class uncertainty. Integrated three-step, pseudo-class, or probability-weighted approaches should be considered in future work. The continuous sensitivity analyses also showed that profile membership did not significantly increase explained variance beyond nonlinear overall VAE, so the retained profiles should be interpreted primarily as a descriptive person-centered representation.

Fourth, participants came from one university context and were recruited by convenience sampling, limiting generalizability. Although sensitivity analyses adjusted for age, gender, academic year, disciplinary area, previous formal visual-arts training, perceived family economic status, and leisure time, with extended models also considering recent academic stress and prior counseling or psychological treatment, these adjustments cannot eliminate confounding. Broader socioeconomic circumstances, academic performance, previous mental-health difficulties, personality, family context, and other unmeasured factors may still influence both arts engagement and mental health. Residual and unmeasured confounding therefore remains a plausible explanation for part of the observed profile differences. Replication across institutions, regions, disciplines, and more diverse student populations is needed.

Fifth, VAE was operationalized primarily through participation frequency across broad domains. Although frequency provided a consistent metric for comparing levels of participation, it did not capture the full conceptual breadth of engagement, including its quality, motivation, emotional meaning, or social context. Future research should directly assess students’ motives for engaging in visual arts, the emotional functions served by participation, and the perceived quality or meaning of the engagement experience. Such measures would help determine whether similar levels of participation reflect enjoyment, self-expression, social connection, coping with distress, avoidance, emotional release, or other psychologically distinct forms of engagement. Additional distinctions, such as online versus offline and solitary versus social engagement, may also help identify qualitatively different configurations that were not evident in the present level-based profiles.

Finally, the study focused on CR and ES, which were themselves strongly correlated. Emotion regulation is broader and more dynamic than these two habitual strategies. Future work should include rumination, acceptance, mindfulness, emotional clarity, regulatory flexibility, and emotion-regulation goals, preferably using methods that reduce shared self-report variance and capture regulation in context.

## 8 Conclusion

In conclusion, visual arts engagement among Chinese non-art-major university students was represented by four empirically derived levels ranging from very low to high engagement. In this cross-sectional sample, the high-engagement profile showed lower subjective well-being and higher depression, anxiety, stress, and overall psychological distress than the lower-engagement profiles. At the same time, continuous linear, quadratic, and spline sensitivity analyses indicated that profile membership did not provide substantial incremental prediction beyond overall VAE, reinforcing interpretation of the profiles as a person-centered description of a graded continuum rather than discrete psychological types. Emotion-regulation variables were statistically involved in the observed associations, but the substantial overlap between cognitive reappraisal and expressive suppression precludes strong strategy-specific conclusions about their distinct relative contributions. These findings describe covariation rather than temporal or causal effects. Future research should therefore move beyond participation frequency to test the motivation, context, quality, and emotional function of visual arts engagement using longitudinal and experimental designs. Such work is necessary before specific campus visual arts practices can be recommended as mental-health interventions.

## Data Availability

The raw data supporting the conclusions of this article will be made available by the authors, without undue reservation.

## References

[ref1] Aldao A. Nolen-Hoeksema S. Schweizer S. (2010). Emotion-regulation strategies across psychopathology: a meta-analytic review. Clin. Psychol. Rev. 30, 217–237. doi: 10.1016/j.cpr.2009.11.004, 20015584

[ref2] Alonso J. Mortier P. Auerbach R. P. Bruffaerts R. Vilagut G. Cuijpers P. . (2018). Severe role impairment associated with mental disorders: results of the WHO world mental health surveys international college student project. Depress. Anxiety 35, 802–814. doi: 10.1002/da.22778, 29847006 PMC6123270

[ref3] Appel H. Gerlach A. L. Crusius J. (2016). The interplay between Facebook use, social comparison, envy, and depression. Curr. Opin. Psychol. 9, 44–49. doi: 10.1016/j.copsyc.2015.10.006

[ref4] Arnett J. J. (2000). Emerging adulthood: a theory of development from the late teens through the twenties. Am. Psychol. 55, 469–480. doi: 10.1037/0003-066X.55.5.469, 10842426

[ref5] Auerbach R. P. Mortier P. Bruffaerts R. Alonso J. Benjet C. Cuijpers P. . (2018). The WHO world mental health surveys international college student project: prevalence and distribution of mental disorders. J. Abnorm. Psychol. 127, 623–638. doi: 10.1037/abn0000362 PMC6193834 30211576

[ref6] Bone J. K. Bu F. Fluharty M. E. Paul E. Sonke J. K. Fancourt D. (2021). Who engages in the arts in the United States? A comparison of several types of engagement using data from the general social survey. BMC Public Health 21:1349. doi: 10.1186/s12889-021-11263-0, 34238255 PMC8264486

[ref7] Butler E. A. Lee T. L. Gross J. J. (2007). Emotion regulation and culture: are the social consequences of emotion suppression culture-specific? Emotion 7, 30–48. doi: 10.1037/1528-3542.7.1.30, 17352561

[ref8] Chen Y. Zeng X. Tao L. Chen J. Wang Y. (2022). The influence of arts engagement on the mental health of isolated college students during the COVID-19 outbreak in China. Front. Public Health 10:1021642. doi: 10.3389/fpubh.2022.1021642, 36457314 PMC9706106

[ref9] Conley C. S. Kirsch A. C. Dickson D. A. Bryant F. B. (2014). Negotiating the transition to college: developmental trajectories and gender differences in psychological functioning, cognitive-affective strategies, and social well-being. Emerg. Adulthood 2, 195–210. doi: 10.1177/2167696814521808

[ref10] Conley C. S. Shapiro J. B. Huguenel B. M. Kirsch A. C. (2020). Navigating the college years: developmental trajectories and gender differences in psychological functioning, cognitive-affective strategies, and social well-being. Emerg. Adulthood 8, 103–117. doi: 10.1177/2167696818791603

[ref11] Cotter K. N. Pawelski J. O. (2022). Art museums as institutions for human flourishing. J. Posit. Psychol. 17, 288–302. doi: 10.1080/17439760.2021.2016911

[ref12] Davies C. Knuiman M. Rosenberg M. (2016). The art of being mentally healthy: a study to quantify the relationship between recreational arts engagement and mental well-being in the general population. BMC Public Health 16:15. doi: 10.1186/s12889-015-2672-7 PMC4702355 26733272

[ref13] Davies C. R. Rosenberg M. Knuiman M. Ferguson R. Pikora T. Slatter N. (2012). Defining arts engagement for population-based health research: art forms, activities and level of engagement. Arts Health 4, 203–216. doi: 10.1080/17533015.2012.656201

[ref14] Delacre M. Leys C. Mora Y. L. Lakens D. (2019). Taking parametric assumptions seriously: arguments for the use of Welch’s *F*-test instead of the classical F-test in one-way ANOVA. Int. Rev. Soc. Psychol. 32:13. doi: 10.5334/irsp.198

[ref15] Drake J. E. Papazian K. Grossman E. (2024). Gravitating toward the arts during the COVID-19 pandemic. Psychol. Aesthet. Creat. Arts 18, 654–665. doi: 10.1037/aca0000471

[ref16] Drake J. E. Winner E. (2012). Confronting sadness through art-making: distraction is more beneficial than venting. Psychol. Aesthet. Creat. Arts 6, 255–261. doi: 10.1037/a0026909

[ref17] Fan J. Ni X. Wu T. Wang Y. Qian Y. (2024). Psychological benefits of arts participation for emerging adulthood: a pathway to flourishing. Behav Sci 14:448. doi: 10.3390/bs14060448, 38920780 PMC11200805

[ref18] Fancourt D. Finn S. (2019). What Is the Evidence on the Role of the Arts in Improving Health and Well-Being? A Scoping Review (Health Evidence Network Synthesis Report 67). Copenhagen: WHO Regional Office for Europe. 32091683

[ref19] Fancourt D. Garnett C. Spiro N. West R. Müllensiefen D. (2019). How do artistic creative activities regulate our emotions? Validation of the emotion regulation strategies for artistic creative activities scale (ERS-ACA). PLoS One 14:e0211362. doi: 10.1371/journal.pone.0211362, 30721269 PMC6363280

[ref20] Fancourt D. Stringaris A. Sacco P. L. (2026). Mechanisms underpinning the mental health impact of arts engagement. Nat. Rev. Psychol. 5, 290–302. doi: 10.1038/s44159-026-00545-2

[ref21] Fung S.-F. Kong C. Y. W. Liu Y.-M. Huang Q. Xiong Z. Jiang Z. . (2022). Validity and psychometric evaluation of the Chinese version of the 5-item WHO well-being index. Front. Public Health 10:872436. doi: 10.3389/fpubh.2022.872436, 35433612 PMC9005828

[ref22] Games P. A. Howell J. F. (1976). Pairwise multiple comparison procedures with unequal n’s and/or variances: a Monte Carlo study. J. Educ. Stat. 1, 113–125. doi: 10.3102/10769986001002113, 38293548

[ref23] Gross J. J. (1998). The emerging field of emotion regulation: an integrative review. Rev. Gen. Psychol. 2, 271–299. doi: 10.1037/1089-2680.2.3.271

[ref24] Gross J. J. (2015a). Emotion regulation: current status and future prospects. Psychol. Inq. 26, 1–26. doi: 10.1080/1047840X.2014.940781

[ref25] Gross J. J. (2015b). The extended process model of emotion regulation: elaborations, applications, and future directions. Psychol. Inq. 26, 130–137. doi: 10.1080/1047840X.2015.989751

[ref26] Gross J. J. John O. P. (2003). Individual differences in two emotion regulation processes: implications for affect, relationships, and well-being. J. Pers. Soc. Psychol. 85, 348–362. doi: 10.1037/0022-3514.85.2.348, 12916575

[ref27] Gruber H. Oepen R. (2018). Emotion regulation strategies and effects in art-making: a narrative synthesis. Arts Psychother. 59, 65–74. doi: 10.1016/j.aip.2017.12.006

[ref28] Hayes A. F. Preacher K. J. (2014). Statistical mediation analysis with a multicategorical independent variable. Br. J. Math. Stat. Psychol. 67, 451–470. doi: 10.1111/bmsp.12028, 24188158

[ref29] Howard M. C. Hoffman M. E. (2018). Variable-centered, person-centered, and person-specific approaches: where theory meets the method. Organ. Res. Methods 21, 846–876. doi: 10.1177/1094428117744021

[ref30] Keyes C. L. M. (2005). Mental illness and/or mental health? Investigating axioms of the complete state model of health. J. Consult. Clin. Psychol. 73, 539–548. doi: 10.1037/0022-006X.73.3.539, 15982151

[ref31] Krause A. E. North A. C. Davidson J. W. (2019). Using self-determination theory to examine musical participation and well-being. Front. Psychol. 10:Article 405. doi: 10.3389/fpsyg.2019.00405, 30881330 PMC6407371

[ref32] Li C.-H. Wu J.-J. (2020). Psychometric evaluation of the Chinese version of the emotion regulation questionnaire in Taiwanese college students. Assessment 27, 1300–1309. doi: 10.1177/1073191118773875, 29749257

[ref33] Lincoln T. M. Schulze L. Renneberg B. (2022). The role of emotion regulation in the characterization, development and treatment of psychopathology. Nat. Rev. Psychol. 1, 272–286. doi: 10.1038/s44159-022-00040-4

[ref34] Lovibond S. H. Lovibond P. F. (1995). Manual for the Depression Anxiety Stress Scales. 2nd Edn. Sydney: Psychology Foundation.

[ref35] Lu S. Hu S. Guan Y. Xiao J. Cai D. Gao Z. . (2018). Measurement invariance of the depression anxiety stress Scales-21 across gender in a sample of Chinese university students. Front. Psychol. 9:2064. doi: 10.3389/fpsyg.2018.02064, 30429809 PMC6220040

[ref36] Mak H. W. Hu Y. Bu F. Bone J. K. Fancourt D. (2024). Art for health’s sake or health for art’s sake: disentangling the bidirectional relationships between arts engagement and mental health. PNAS Nexus 3:pgae465. doi: 10.1093/pnasnexus/pgae465, 39588318 PMC11586668

[ref37] Markus H. R. Kitayama S. (1991). Culture and the self: implications for cognition, emotion, and motivation. Psychol. Rev. 98, 224–253. doi: 10.1037/0033-295X.98.2.224

[ref38] Masyn K. E. (2013). “Latent class analysis and finite mixture modeling,” in The Oxford Handbook of Quantitative Methods in Psychology: Vol. 2: Statistical Analysis, ed. Little T. D. (New York: Oxford University Press), 551–611.

[ref39] Moussa M. T. Lovibond P. F. Laube R. (2001). Psychometric Properties of a Chinese Version of the Short Depression Anxiety Stress Scales (DASS21). Report for New South Wales Transcultural Mental Health Centre, Cumberland Hospital. Sydney.

[ref40] Nylund K. L. Asparouhov T. Muthén B. O. (2007). Deciding on the number of classes in latent class analysis and growth mixture modeling: a Monte Carlo simulation study. Struct. Equ. Model. 14, 535–569. doi: 10.1080/10705510701575396

[ref41] Nylund-Gibson K. Choi A. Y. (2018). Ten frequently asked questions about latent class analysis. Transl. Issues Psychol. Sci. 4, 440–461. doi: 10.1037/tps0000176

[ref42] Preacher K. J. Hayes A. F. (2008). Asymptotic and resampling strategies for assessing and comparing indirect effects in multiple mediator models. Behav. Res. Methods 40, 879–891. doi: 10.3758/BRM.40.3.879, 18697684

[ref43] Ren K. Pang C. Sun J. Xie Z. (2026). The (in)congruence between creative family and school environments and Chinese adolescents’ creative self-efficacy: the mediating role of growth mindset. Think. Skills Creat. 62:102317. doi: 10.1016/j.tsc.2026.102317

[ref44] Rodriguez A. K. Akram S. Colverson A. J. Hack G. Golden T. L. Sonke J. (2024). Arts engagement as a health behavior: an opportunity to address mental health inequities. Community Health Equity Res Policy 44, 315–322. doi: 10.1177/2752535X231175072, 37196338 PMC11409561

[ref45] Rosseel Y. (2012). Lavaan: an R package for structural equation modeling. J. Stat. Softw. 48, 1–36. doi: 10.18637/jss.v048.i02

[ref46] Ryan R. M. Deci E. L. (2000). Self-determination theory and the facilitation of intrinsic motivation, social development, and well-being. Am. Psychol. 55, 68–78. doi: 10.1037/0003-066X.55.1.68, 11392867

[ref47] Shim Y. Jebb A. T. Tay L. Pawelski J. O. (2021). Arts and humanities interventions for flourishing in healthy adults: a mixed studies systematic review. Rev. Gen. Psychol. 25, 258–282. doi: 10.1177/10892680211021350

[ref48] Sonke J. Rodriguez A. K. Colverson A. Akram S. Morgan N. Hancox D. . (2024). Defining “arts participation” for public health research. Health Promot. Pract. 25, 985–996. doi: 10.1177/15248399231183388, 37458132 PMC11528960

[ref49] Tein J.-Y. Coxe S. Cham H. (2013). Statistical power to detect the correct number of classes in latent profile analysis. Struct. Equ. Model. Multidiscip. J. 20, 640–657. doi: 10.1080/10705511.2013.824781, 24489457 PMC3904803

[ref50] Thornton E. Petersen K. Marquez J. Humphrey N. (2024). Do patterns of adolescent participation in arts, culture and entertainment activities predict later wellbeing? A latent class analysis. J. Youth Adolesc. 53, 1396–1414. doi: 10.1007/s10964-024-01950-7, 38466529 PMC11045570

[ref51] Tomlinson A. Lane J. Julier G. Grigsby Duffy L. Payne A. Mansfield L. . (2020). Qualitative findings from a systematic review: visual arts engagement for adults with mental health conditions. J. Appl. Arts Health 11, 281–297. doi: 10.1386/jaah_00042_1

[ref52] Topp C. W. Østergaard S. D. Søndergaard S. Bech P. (2015). The WHO-5 well-being index: a systematic review of the literature. Psychother. Psychosom. 84, 167–176. doi: 10.1159/000376585, 25831962

[ref53] Trupp M. D. Howlin C. Fekete A. Kutsche J. Fingerhut J. Pelowski M. (2025). The impact of viewing art on well-being—a systematic review of the evidence base and suggested mechanisms. J. Posit. Psychol. 20, 978–1002. doi: 10.1080/17439760.2025.2481041

[ref54] Tsai J. L. (2007). Ideal affect: cultural causes and behavioral consequences. Perspect. Psychol. Sci. 2, 242–259. doi: 10.1111/j.1745-6916.2007.00043.x, 26151968

[ref55] Verduyn P. Lee D. S. Park J. Shablack H. Orvell A. Bayer J. B. . (2015). Passive Facebook usage undermines affective well-being: experimental and longitudinal evidence. J. Exp. Psychol. Gen. 144, 480–488. doi: 10.1037/xge0000057, 25706656

[ref56] Wang S. Mak H. W. Fancourt D. (2020). Arts, mental distress, mental health functioning & life satisfaction: fixed-effects analyses of a nationally-representative panel study. BMC Public Health 20:208. doi: 10.1186/s12889-019-8109-y, 32046670 PMC7014626

[ref57] Wang K. Shi H.-S. Geng F.-L. Zou L.-Q. Tan S.-P. Wang Y. . (2016). Research on translations of tests: cross-cultural validation of the depression anxiety stress Scale-21 in China. Psychol. Assess. 28, e88–e100. doi: 10.1037/pas0000207, 26619091

[ref58] Wei M. Su J. C. Carrera S. Lin S.-P. Yi F. (2013). Suppression and interpersonal harmony: a cross-cultural comparison between Chinese and European Americans. J. Couns. Psychol. 60, 625–633. doi: 10.1037/a0033413, 23978268

[ref59] Welch B. L. (1951). On the comparison of several mean values: an alternative approach. Biometrika 38, 330–336. doi: 10.1093/biomet/38.3-4.330

[ref60] Westerhof G. J. Keyes C. L. M. (2010). Mental illness and mental health: the two continua model across the lifespan. J. Adult Dev. 17, 110–119. doi: 10.1007/s10804-009-9082-y, 20502508 PMC2866965

[ref61] World Health Organization (2024). The World Health Organization-Five Well-Being Index (WHO-5). Available online at: https://www.who.int/publications/m/item/WHO-UCN-MSD-MHE-2024.01 (Accessed August 28, 2026).

