# Supplementary material for: Beyond the more-is-better assumption: latent profiles of visual arts engagement, emotion regulation, and mental health among Chinese non-art-major university students
Source: Front Psychol. 2026 Sep 9;17:1934081. doi: 10.3389/fpsyg.2026.1934081 (PMC13597247; doi:10.3389/fpsyg.2026.1934081)
Supplement: Supplementary file 1 [file Data_Sheet_1.PDF]

Appendix A

Supplementary Materials

Table S1

Full Games–Howell Pairwise Comparisons, Hedges' g, and FDR Sensitivity Results Across the Four VAE Profiles

Variable	Comparison	Mean difference	95% CI	Hedges' g	GH p	BH-FDR p	FDR result	Raw result
Cognitive reappraisal	Very low vs. low	0.243	[-0.162, 0.648]	0.180	.407	.535	Nonsignificant	Nonsignificant
Cognitive reappraisal	Very low vs. moderate	0.798	[0.374, 1.223]	0.578	< .001	< .001	Significant	Significant
Cognitive reappraisal	Very low vs. high	1.659	[1.206, 2.113]	1.172	< .001	< .001	Significant	Significant
Cognitive reappraisal	Low vs. moderate	0.555	[0.163, 0.947]	0.406	.002	.003	Significant	Significant
Cognitive reappraisal	Low vs. high	1.416	[0.993, 1.840]	1.013	< .001	< .001	Significant	Significant
Cognitive reappraisal	Moderate vs. high	0.861	[0.419, 1.304]	0.601	< .001	< .001	Significant	Significant
Expressive suppression	Very low vs. low	0.313	[-0.086, 0.712]	0.232	.181	.262	Nonsignificant	Nonsignificant
Expressive suppression	Very low vs. moderate	0.834	[0.418, 1.249]	0.614	< .001	< .001	Significant	Significant
Expressive suppression	Very low vs. high	1.606	[1.160, 2.051]	1.155	< .001	< .001	Significant	Significant
Expressive suppression	Low vs. moderate	0.521	[0.122, 0.920]	0.373	.005	.008	Significant	Significant
Expressive suppression	Low vs. high	1.293	[0.863, 1.723]	0.907	< .001	< .001	Significant	Significant
Expressive suppression	Moderate vs. high	0.772	[0.327, 1.217]	0.535	< .001	< .001	Significant	Significant
WHO-5 raw score	Very low vs. low	0.892	[-0.755, 2.539]	0.161	.500	.637	Nonsignificant	Nonsignificant
WHO-5 raw score	Very low vs. moderate	3.523	[1.734, 5.312]	0.599	< .001	< .001	Significant	Significant
WHO-5 raw score	Very low vs. high	6.766	[4.889, 8.644]	1.154	< .001	< .001	Significant	Significant
WHO-5 raw score	Low vs. moderate	2.631	[0.928, 4.335]	0.444	< .001	< .001	Significant	Significant

Variable	Comparison	Mean difference	95% CI	Hedges' g	GH p	BH-FDR p	FDR result	Raw result
WHO-5 raw score	Low vs. high	5.874	[4.077, 7.671]	0.993	< .001	< .001	Significant	Significant
WHO-5 raw score	Moderate vs. high	3.243	[1.316, 5.170]	0.518	< .001	< .001	Significant	Significant
Depression	Very low vs. low	0.253	[-1.330, 1.836]	0.049	.976	.998	Nonsignificant	Nonsignificant
Depression	Very low vs. moderate	-0.850	[-2.554, 0.855]	-0.153	.571	.685	Nonsignificant	Nonsignificant
Depression	Very low vs. high	-2.964	[-4.794, -1.134]	-0.519	< .001	< .001	Significant	Significant
Depression	Low vs. moderate	-1.103	[-2.618, 0.413]	-0.209	.239	.335	Nonsignificant	Nonsignificant
Depression	Low vs. high	-3.217	[-4.873, -1.561]	-0.595	< .001	< .001	Significant	Significant
Depression	Moderate vs. high	-2.114	[-3.886, -0.342]	-0.369	.012	.019	Significant	Significant
Anxiety	Very low vs. low	0.510	[-0.928, 1.948]	0.108	.796	.880	Nonsignificant	Nonsignificant
Anxiety	Very low vs. moderate	-0.104	[-1.630, 1.422]	-0.021	.998	.998	Nonsignificant	Nonsignificant
Anxiety	Very low vs. high	-2.668	[-4.357, -0.978]	-0.506	< .001	< .001	Significant	Significant
Anxiety	Low vs. moderate	-0.614	[-1.974, 0.746]	-0.130	.649	.757	Nonsignificant	Nonsignificant
Anxiety	Low vs. high	-3.178	[-4.720, -1.635]	-0.634	< .001	< .001	Significant	Significant
Anxiety	Moderate vs. high	-2.564	[-4.188, -0.940]	-0.490	< .001	< .001	Significant	Significant
Stress	Very low vs. low	0.291	[-1.342, 1.925]	0.054	.967	.998	Nonsignificant	Nonsignificant
Stress	Very low vs. moderate	-0.901	[-2.636, 0.833]	-0.161	.536	.662	Nonsignificant	Nonsignificant
Stress	Very low vs. high	-2.963	[-4.872, -1.054]	-0.497	< .001	< .001	Significant	Significant
Stress	Low vs. moderate	-1.193	[-2.699, 0.314]	-0.227	.174	.261	Nonsignificant	Nonsignificant
Stress	Low vs. high	-3.254	[-4.961, -1.547]	-0.587	< .001	< .001	Significant	Significant
Stress	Moderate vs. high	-2.061	[-3.865, -0.258]	-0.355	.018	.028	Significant	Significant
Overall PD	Very low vs. low	1.054	[-3.417, 5.525]	0.072	.929	.998	Nonsignificant	Nonsignificant
Overall PD	Very low vs. moderate	-1.855	[-6.638, 2.928]	-0.120	.748	.849	Nonsignificant	Nonsignificant
Overall PD	Very low vs. high	-8.595	[-13.844, -3.346]	-0.524	< .001	< .001	Significant	Significant
Overall PD	Low vs. moderate	-2.909	[-7.107, 1.290]	-0.199	.280	.380	Nonsignificant	Nonsignificant
Overall PD	Low vs. high	-9.649	[-14.375, -4.922]	-0.629	< .001	< .001	Significant	Significant
Overall PD	Moderate vs. high	-6.740	[-11.762, -1.718]	-0.416	.003	.006	Significant	Significant

Note. VAE = visual arts engagement; PD = psychological distress; GH = Games–Howell. Mean differences and Hedges' g follow the comparison order (first group minus second group). BH-FDR p values were calculated across the full set of pairwise tests as a multiplicity sensitivity analysis.

Table S2

BCH-Adjusted Distal Outcome Means Across the Four VAE Profiles

Outcome	Very low VAE	Low VAE	Moderate VAE	High VAE	BCH overall test
Cognitive reappraisal	5.241	4.980	4.423	3.560	$\chi^2(3) = 109.392$, $p < .001$
Expressive suppression	5.236	4.900	4.381	3.607	$\chi^2(3) = 100.712$, $p < .001$
WHO-5 raw score	17.672	16.720	14.069	10.827	$\chi^2(3) = 107.030$, $p < .001$
Depression	7.680	7.396	8.530	10.653	$\chi^2(3) = 27.924$, $p < .001$
Anxiety	8.196	7.636	8.280	10.866	$\chi^2(3) = 30.146$, $p < .001$
Stress	7.852	7.526	8.754	10.822	$\chi^2(3) = 26.702$, $p < .001$
Overall psychological distress	23.728	22.558	25.564	32.341	$\chi^2(3) = 29.893$, $p < .001$

Note. VAE = visual arts engagement; BCH = Bolck–Croon–Hagenaars method. Values are BCH-adjusted distal-outcome means based on the corrected WHO-5 and DASS-21 scores. The BCH overall test accounts for classification uncertainty.

Table S3

Class-Specific Indicator Means, Classification Characteristics, and Common Indicator Variances for the Three-, Four-, and Five-Profile Solutions

Panel A. Class-specific means and classification characteristics

Solution	Ordered profile	Mplus class	Modal n (%)	APP	AC M (SE)	RA M (SE)	DE M (SE)	SI M (SE)	Average
3-profile	Profile 1	1	298 (50.85%)	0.976	1.665 (0.035)	1.738 (0.035)	1.772 (0.035)	1.751 (0.032)	1.732
3-profile	Profile 2	2	158 (26.96%)	0.988	2.969 (0.037)	3.009 (0.040)	2.951 (0.041)	2.963 (0.046)	2.973
3-profile	Profile 3	3	130 (22.18%)	0.989	4.233 (0.046)	4.228 (0.049)	4.212 (0.048)	4.212 (0.048)	4.221
4-profile	Very low VAE	1	129 (22.01%)	0.954	1.230 (0.031)	1.350 (0.037)	1.372 (0.046)	1.401 (0.038)	1.338
4-profile	Low VAE	2	174 (29.69%)	0.957	2.040 (0.034)	2.080 (0.036)	2.107 (0.036)	2.049 (0.033)	2.069
4-profile	Moderate VAE	4	153 (26.11%)	0.983	3.010 (0.031)	3.049 (0.036)	3.002 (0.035)	3.018 (0.040)	3.020
4-profile	High VAE	3	130 (22.18%)	0.989	4.240 (0.046)	4.230 (0.049)	4.215 (0.049)	4.213 (0.048)	4.224
5-profile	Profile 1	1	128 (21.84%)	0.967	1.226 (0.033)	1.343 (0.038)	1.380 (0.047)	1.396 (0.039)	1.336
5-profile	Profile 2	3	175 (29.86%)	0.962	2.042 (0.033)	2.082 (0.036)	2.101 (0.036)	2.051 (0.033)	2.069
5-profile	Profile 3	2	151 (25.77%)	0.982	3.000 (0.031)	3.038 (0.036)	2.986 (0.035)	2.999 (0.038)	3.006
5-profile	Profile 4	5	85 (14.51%)	0.965	3.985 (0.048)	3.938 (0.048)	3.998 (0.051)	3.946 (0.050)	3.967
5-profile	Profile 5	4	47 (8.02%)	0.945	4.640 (0.080)	4.715 (0.059)	4.565 (0.100)	4.677 (0.059)	4.649

Panel B. Indicator variances constrained equal across profiles within each solution

Solution	Indicator	Variance	SE	z	p
3-profile	AC	0.237	0.013	18.312	< .001
3-profile	DE	0.272	0.016	16.728	< .001
3-profile	RA	0.260	0.014	18.056	< .001
3-profile	SI	0.257	0.015	16.992	< .001
4-profile	AC	0.158	0.013	12.182	< .001
4-profile	DE	0.201	0.017	11.763	< .001
4-profile	RA	0.200	0.015	13.661	< .001
4-profile	SI	0.202	0.015	13.356	< .001
5-profile	AC	0.138	0.015	9.310	< .001
5-profile	DE	0.188	0.020	9.490	< .001
5-profile	RA	0.167	0.015	11.066	< .001
5-profile	SI	0.168	0.015	11.229	< .001

Note. APP = average posterior probability; AC = active creation; RA = receptive appreciation; DE = digital engagement; SI = social/learning involvement. Class-specific indicator means were freely estimated; indicator variances were constrained equal across profiles within a solution; within-profile covariances were fixed to zero. The best log-likelihood was replicated for the three-, four-, and five-profile solutions.

Table S4

Descriptive Distributional Statistics and Zero-Order Correlations Among the Main Study Variables

Panel A. Descriptive distributional statistics

Variable	M	SD	Observed range	Skewness	Kurtosis	Floor %	Ceiling %	α
AC	2.60	1.14	1–5	0.344	-0.846	13.48	3.75	0.890
RA	2.65	1.12	1–5	0.372	-0.817	8.36	4.27	0.872
DE	2.64	1.11	1–5	0.353	-0.810	9.22	2.73	0.863
SI	2.64	1.11	1–5	0.417	-0.745	8.36	3.58	0.865
VAE total	2.63	1.06	1–5	0.410	-0.888	0.51	0.17	0.964
CR	4.58	1.51	1–7	-0.369	-0.652	0.85	3.41	0.948
ES	4.55	1.50	1–7	-0.302	-0.726	1.02	4.27	0.908
WHO-5 raw score	14.94	6.39	0–25	-0.438	-0.742	1.02	2.90	0.913
Depression	8.47	5.58	0–21	0.391	-0.829	4.10	1.71	0.908
Anxiety	8.64	5.10	0–21	0.318	-0.888	1.02	0.34	0.856
Stress	8.64	5.68	0–21	0.375	-0.856	2.90	1.02	0.917
Overall PD	25.75	15.77	1–61	0.397	-0.931	0.00	0.00	0.962

Panel B. Pearson zero-order correlation matrix

Variable	1	2	3	4	5	6	7	8	9	10	11	12
1. AC	—											
2. RA	0.881	—										
3. DE	0.870	0.857	—									
4. SI	0.871	0.856	0.849	—								
5. VAE total	0.956	0.948	0.943	0.943	—							
6. CR	-0.380	-0.364	-0.360	-0.386	-0.393	—						
7. ES	-0.369	-0.358	-0.353	-0.369	-0.383	0.910	—					
8. WHO-5	-0.364	-0.358	-0.363	-0.385	-0.388	0.882	0.856	—				
9. Depression	0.188	0.189	0.180	0.197	0.199	-0.291	-0.250	-0.282	—			
10. Anxiety	0.171	0.173	0.174	0.196	0.188	-0.279	-0.238	-0.257	0.888	—		
11. Stress	0.189	0.191	0.188	0.215	0.206	-0.295	-0.277	-0.284	0.910	0.879	—	
12. Overall PD	0.190	0.192	0.188	0.211	0.206	-0.300	-0.265	-0.285	0.969	0.955	0.967	—

Note. N = 586. AC = active creation; RA = receptive appreciation; DE = digital engagement; SI = social/learning involvement; VAE = visual arts engagement; CR = cognitive reappraisal; ES = expressive suppression; WHO-5 = World Health Organization-Five Well-Being Index; PD = psychological distress. Floor and ceiling percentages use the theoretical minimum and maximum of each score. All off-diagonal Pearson correlations shown were $p < .001$.

Table S5

Continuous VAE Sensitivity Analyses and Incremental Tests of Profile Membership

Panel A. Model comparison for the two primary mental-health outcomes

Outcome	Model	R^2	Adjusted R^2	AIC	BIC	Quadratic B (p)
WHO-5 subjective well-being	Continuous linear	0.150	0.149	3746.56	3759.68	—
WHO-5 subjective well-being	Continuous quadratic	0.151	0.148	3748.06	3765.55	-0.177 (.481)
WHO-5 subjective well-being	Continuous spline (df=3)	0.154	0.149	3748.33	3770.20	—
WHO-5 subjective well-being	Profile only	0.157	0.153	3745.64	3767.51	—
WHO-5 subjective well-being	Quadratic + profile	0.158	0.151	3749.24	3779.85	-0.177 (.481)
WHO-5 subjective well-being	Spline + profile	0.158	0.149	3751.19	3786.18	—
Overall psychological distress	Continuous linear	0.042	0.041	4874.81	4887.93	—
Overall psychological distress	Continuous quadratic	0.051	0.048	4871.52	4889.01	1.502 (.022)
Overall psychological distress	Continuous spline (df=3)	0.052	0.047	4872.76	4894.63	—
Overall psychological distress	Profile only	0.054	0.049	4871.87	4893.73	—
Overall psychological distress	Quadratic + profile	0.055	0.047	4874.96	4905.57	1.502 (.022)
Overall psychological distress	Spline + profile	0.055	0.045	4876.85	4911.84	—

Panel B. Nonlinearity and incremental profile-membership tests across all outcomes

Outcome	Test	df added	F	p	ΔR^2	BH-FDR p
Cognitive reappraisal	Quadratic term beyond linear VAE	1	1.588	.208	0.002	.437
Cognitive reappraisal	Profile membership beyond quadratic VAE	3	2.028	.109	0.009	.437
Cognitive reappraisal	Profile membership beyond spline VAE	3	1.649	.177	0.007	.437
Expressive suppression	Quadratic term beyond linear VAE	1	0.346	.556	0.001	.656
Expressive suppression	Profile membership beyond quadratic VAE	3	0.941	.420	0.004	.656
Expressive suppression	Profile membership beyond spline VAE	3	0.713	.544	0.003	.656
WHO-5 subjective well-being	Quadratic term beyond linear VAE	1	0.497	.481	0.001	.656
WHO-5 subjective well-being	Profile membership beyond quadratic VAE	3	1.599	.189	0.007	.437
WHO-5 subjective well-being	Profile membership beyond spline VAE	3	1.038	.375	0.005	.656
Depression	Quadratic term beyond linear VAE	1	3.899	.049	0.006	.256
Depression	Profile membership beyond quadratic VAE	3	0.941	.420	0.005	.656
Depression	Profile membership beyond spline VAE	3	0.684	.562	0.003	.656
Anxiety	Quadratic term beyond linear VAE	1	7.455	.007	0.012	.137

Outcome	Test	df added	F	p	ΔR²	BH-FDR p
Anxiety	Profile membership beyond quadratic VAE	3	1.823	.142	0.009	.437
Anxiety	Profile membership beyond spline VAE	3	1.523	.208	0.007	.437
Stress	Quadratic term beyond linear VAE	1	3.929	.048	0.006	.256
Stress	Profile membership beyond quadratic VAE	3	0.292	.831	0.001	.873
Stress	Profile membership beyond spline VAE	3	0.212	.888	0.001	.888
Overall psychological distress	Quadratic term beyond linear VAE	1	5.290	.022	0.009	.229
Overall psychological distress	Profile membership beyond quadratic VAE	3	0.846	.469	0.004	.656
Overall psychological distress	Profile membership beyond spline VAE	3	0.629	.596	0.003	.659

Note. The overall VAE score was standardized before continuous-model estimation. Natural splines used 3 degrees of freedom. BH-FDR values provide a sensitivity correction across the nonlinearity and incremental-profile tests. Profile membership did not add significant explanatory value beyond the quadratic or spline specifications for any outcome.

Table S6
Confirmatory Factor Analysis Results Using WLSMV With Ordinal Indicators

Panel A. Model fit

Model	χ^2	df	p	CFI	TLI	RMSEA	SRMR
VAE four-factor	172.35	48	< .001	0.997	0.996	0.067	0.013
ERQ two-factor	105.19	34	< .001	0.998	0.998	0.060	0.007
WHO-5 one-factor	14.87	5	.011	0.999	0.997	0.058	0.010
DASS-21 three-factor	331.88	186	< .001	0.997	0.996	0.037	0.019

Table S6 (continued). Panel B. Standardized factor loadings

Model	Factor	Item	Std. loading	SE	z	p	95% CI lower	95% CI upper
VAE four-factor	AC	ac1	1.004	0.004	241.02	< .001	0.996	1.012
VAE four-factor	AC	ac2	0.810	0.015	54.67	< .001	0.781	0.839
VAE four-factor	AC	ac3	0.855	0.012	70.38	< .001	0.831	0.878
VAE four-factor	RA	ra1	0.845	0.013	62.84	< .001	0.819	0.871
VAE four-factor	RA	ra2	0.877	0.011	78.68	< .001	0.855	0.899
VAE four-factor	RA	ra3	0.873	0.011	76.37	< .001	0.851	0.896
VAE four-factor	DE	de1	0.852	0.013	66.36	< .001	0.827	0.877
VAE four-factor	DE	de2	0.847	0.014	62.57	< .001	0.821	0.874
VAE four-factor	DE	de3	0.864	0.013	65.47	< .001	0.838	0.889
VAE four-factor	SI	si1	0.839	0.014	59.29	< .001	0.812	0.867
VAE four-factor	SI	si2	0.849	0.014	62.76	< .001	0.822	0.875
VAE four-factor	SI	si3	0.880	0.012	75.53	< .001	0.857	0.903
ERQ two-factor	CR	erq1	0.999	0.002	435.95	< .001	0.994	1.003
ERQ two-factor	CR	erq3	0.867	0.010	90.05	< .001	0.848	0.886
ERQ two-factor	CR	erq5	0.848	0.011	80.66	< .001	0.828	0.869
ERQ two-factor	CR	erq7	0.845	0.010	81.87	< .001	0.825	0.866
ERQ two-factor	CR	erq8	0.877	0.009	97.97	< .001	0.860	0.895
ERQ two-factor	CR	erq10	0.882	0.009	99.24	< .001	0.864	0.899
ERQ two-factor	ES	erq2	0.871	0.010	88.68	< .001	0.852	0.890
ERQ two-factor	ES	erq4	0.859	0.011	80.50	< .001	0.838	0.880
ERQ two-factor	ES	erq6	0.864	0.010	86.66	< .001	0.845	0.884
ERQ two-factor	ES	erq9	0.852	0.010	81.30	< .001	0.831	0.873
WHO-5 one-factor	WHO	who1	0.838	0.014	58.21	< .001	0.810	0.866
WHO-5 one-factor	WHO	who2	0.855	0.013	63.38	< .001	0.828	0.881
WHO-5 one-factor	WHO	who3	0.825	0.016	50.11	< .001	0.792	0.857
WHO-5 one-factor	WHO	who4	0.853	0.013	65.60	< .001	0.828	0.879
WHO-5 one-factor	WHO	who5_item	0.848	0.015	58.40	< .001	0.819	0.876
DASS-21 three-factor	DEP	dass3	0.793	0.018	43.64	< .001	0.758	0.829
DASS-21 three-factor	DEP	dass5	0.821	0.016	50.76	< .001	0.789	0.853

Model	Factor	Item	Std. loading	SE	z	p	95% CI lower	95% CI upper
DASS-21 three-factor	DEP	dass10	0.831	0.016	52.87	< .001	0.800	0.861
DASS-21 three-factor	DEP	dass13	0.806	0.018	45.87	< .001	0.772	0.840
DASS-21 three-factor	DEP	dass16	0.848	0.014	59.01	< .001	0.820	0.876
DASS-21 three-factor	DEP	dass17	0.797	0.018	44.40	< .001	0.762	0.832
DASS-21 three-factor	DEP	dass21	0.784	0.019	40.24	< .001	0.746	0.822
DASS-21 three-factor	ANX	dass2	0.018	0.045	0.41	.685	-0.071	0.107
DASS-21 three-factor	ANX	dass4	0.859	0.014	60.46	< .001	0.831	0.886
DASS-21 three-factor	ANX	dass7	0.840	0.015	54.35	< .001	0.810	0.871
DASS-21 three-factor	ANX	dass9	0.806	0.016	49.01	< .001	0.774	0.838
DASS-21 three-factor	ANX	dass15	0.824	0.016	50.70	< .001	0.792	0.856
DASS-21 three-factor	ANX	dass19	0.817	0.016	49.78	< .001	0.785	0.849
DASS-21 three-factor	ANX	dass20	0.825	0.016	50.86	< .001	0.793	0.857
DASS-21 three-factor	STR	dass1	1.014	0.004	248.69	< .001	1.006	1.022
DASS-21 three-factor	STR	dass6	0.781	0.019	41.28	< .001	0.744	0.818
DASS-21 three-factor	STR	dass8	0.791	0.017	45.26	< .001	0.757	0.825
DASS-21 three-factor	STR	dass11	0.812	0.016	49.47	< .001	0.780	0.844
DASS-21 three-factor	STR	dass12	0.820	0.016	51.27	< .001	0.789	0.852
DASS-21 three-factor	STR	dass14	0.761	0.020	38.85	< .001	0.723	0.799
DASS-21 three-factor	STR	dass18	0.822	0.016	52.49	< .001	0.791	0.852

Panel C. Latent-factor correlations

Model	Factor 1	Factor 2	Std. correlation	SE	z	p
VAE four-factor	AC	RA	0.989	0.005	208.06	< .001
VAE four-factor	AC	DE	0.985	0.005	180.59	< .001
VAE four-factor	AC	SI	0.992	0.006	178.49	< .001
VAE four-factor	RA	DE	0.986	0.007	148.47	< .001
VAE four-factor	RA	SI	0.988	0.007	149.10	< .001
VAE four-factor	DE	SI	0.982	0.006	151.44	< .001
ERQ two-factor	CR	ES	0.981	0.003	300.68	< .001
DASS-21 three-factor	DEP	ANX	0.996	0.005	186.43	< .001
DASS-21 three-factor	DEP	STR	0.996	0.004	281.56	< .001

Model	Factor 1	Factor 2	Std. correlation	SE	z	p
DASS-21 three-factor	ANX	STR	0.987	0.004	251.55	< .001

Note. All CFA models used WLSMV and treated questionnaire indicators as ordered categorical. The high VAE factor correlations indicate substantial common-factor overlap and support cautious interpretation of the LPA as predominantly level-based. The corrected DASS-21 item-level data were used for the reported DASS CFA.

Table S7
Full Visual Arts Engagement Item Set and Analytic Role

Item	Dimension	Analyticrole	Englishwording	OriginalChinesewording	IncludedinLPA
VAE1	AC	Core LPA item	I engage in drawing, sketching, illustration, doodling, or other visual creative activities.	我会进行绘画、素描、插画、涂鸦或其他视觉创作活动	Yes
VAE2	AC	Core LPA item	I use photography, image-making, or visual expression to present my ideas, emotions, or aesthetic preferences.	我会通过摄影、图像制作或视觉表达来呈现自己的想法、情绪或审美	Yes
VAE3	AC	Core LPA item	I make handcrafts with visual design or form elements, such as collage, decoration, ceramics, or other handmade creations.	我会做带有视觉设计或造型成分的手工创作，如拼贴、装饰、陶艺、手作等	Yes
VAE4	RA	Core LPA item	I visit art museums, galleries, exhibitions, or other visual-arts-related events.	我会参观美术馆、画廊、展览或其他与视觉艺术相关的活动	Yes
VAE5	RA	Core LPA item	I actively appreciate paintings, photography, design, sculpture, or other visual artworks.	我会主动欣赏绘画、摄影、设计、雕塑或其他视觉艺术作品	Yes
VAE6	RA	Core LPA item	I notice street art, public art, architecture, or visual-art elements in everyday environments.	我会留意街头艺术、公共艺术、建筑或日常环境中的视觉艺术元素	Yes
VAE7	DE	Core LPA item	I browse visual-arts-related content on social media or online platforms.	我会在社交媒体或网络平台浏览视觉艺术相关内容	Yes
VAE8	DE	Core LPA item	I save, share, repost, or discuss visual artworks that I like.	我会保存、分享、转发或讨论自己喜欢的视觉艺术作品	Yes
VAE9	DE	Core LPA item	I use online courses, tutorials, or accounts to learn visual-arts-related knowledge or skills.	我会通过网络课程、教程或账号学习视觉艺术相关知识或技能	Yes
VAE10	SI	Core LPA item	I participate in visual-arts-related courses, clubs, workshops, or experiential activities.	我会参加与视觉艺术相关的课程、社团、工作坊或体验活动	Yes
VAE11	SI	Core LPA item	I create visual work with others, or show my work to others and receive feedback.	我会和他人一起进行视觉创作，或向别人展示自己的作品并获得反馈	Yes
VAE12	SI	Core LPA item	Even though I am not an art-major student, I am willing to actively try visual arts activities.	即使我不是艺术类专业学生，我也愿意主动尝试视觉艺术活动	Yes
VAE13	—	Supplementary contextual item	During the past three months, approximately how much time per week did you spend on visual-arts-related activities?	在过去 3 个月中，我平均每周花在视觉艺术相关活动上的时间是	No
VAE14	—	Supplementary contextual item	During the past three months, what was your overall frequency of participation in visual arts activities?	在过去 3 个月中，我参与视觉艺术活动的总体频率是	No

Note. AC = active creation; RA = receptive appreciation; DE = digital engagement; SI = social/learning involvement. The 12 core items were rated for engagement during the past three months and were averaged within their prespecified three-item dimensions. VAE13 and VAE14 were retained as contextual supplementary items but were not used as LPA indicators because they did not map directly onto the four prespecified dimensions.

Syntax S1. Mplus Syntax for the Retained Four-Profile LPA

The three- and five-profile comparison models used the same specification, with only the CLASSES statement changed to c(3) or c(5), respectively.

```
TITLE:
  Four-profile latent profile analysis;

DATA:
  FILE IS LPA_3to5classes.dat;

VARIABLE:
  NAMES ARE id ac ra de si;
  USEVARIABLES ARE ac ra de si;
  IDVARIABLE IS id;
  MISSING ARE ALL (-9999);
  CLASSES = c(4);

ANALYSIS:
  TYPE = MIXTURE;
  ESTIMATOR = MLR;
  STARTS = 2000 500;
  STITERATIONS = 20;
  LRTSTARTS = 0 0 500 100;
  PROCESSORS = 4;

OUTPUT:
  TECH1 TECH4 TECH8 TECH11 TECH14;
  SAMPSTAT;

SAVEDATA:
  FILE IS LPA_4class_cprob.dat;
  SAVE = CPROBABILITIES;

PLOT:
  TYPE = PLOT3;
  SERIES = ac(1) ra(2) de(3) si(4);
```

Note. Under this Mplus parameterization, class-specific indicator means were freely estimated, indicator variances were constrained equal across profiles, and within-profile covariances were fixed to zero.

Syntax S2. Mplus Syntax for the BCH Distal-Outcome Sensitivity Analysis

```
TITLE:
 Final four-class LPA with BCH distal outcome comparisons
 after corrected WHO-5 and DASS-21 scoring;

DATA:
 FILE IS MPLUS_BCH_CORRECTED.dat;

VARIABLE:
 NAMES ARE
  id
  ac ra de si
  cr es
  who5 dep anx str pd;

 USEVARIABLES ARE
  ac ra de si;

 IDVARIABLE IS id;
 MISSING ARE ALL (-9999);

 CLASSES = c(4);

 AUXILIARY =
  cr   (BCH)
  es   (BCH)
  who5  (BCH)
  dep  (BCH)
  anx  (BCH)
  str  (BCH)
  pd   (BCH);

ANALYSIS:
 TYPE = MIXTURE;
 ESTIMATOR = MLR;

 ! 2000 random start sets; 500 carried to final optimization.
 STARTS = 2000 500;
 STITERATIONS = 20;

 ! Used for TECH14 bootstrap likelihood-ratio test.
 LRTSTARTS = 0 0 500 100;

 PROCESSORS = 4;

OUTPUT:
 TECH1 TECH4 TECH8 TECH11 TECH14;
 SAMPSTAT;

SAVEDATA:
 FILE IS lpa_4class_corrected_cprob.dat;
 SAVE = CPROBABILITIES;

PLOT:
 TYPE = PLOT3;
 SERIES =
  ac(1)
  ra(2)
  de(3)
  si(4);
```

Note. The BCH model was rerun after the WHO-5 and DASS-21 response-code audit so that the supplementary distal-outcome means correspond to the corrected scoring used throughout the revised manuscript.
